# Optimisation of growth conditions for ovine airway epithelial cell differentiation at an air-liquid interface

**DOI:** 10.1371/journal.pone.0193998

**Published:** 2018-03-08

**Authors:** Nicky O’Boyle, Erin Sutherland, Catherine C. Berry, Robert L. Davies

**Affiliations:** 1 Institute of Infection, Immunity and Inflammation, College of Medical, Veterinary and Life Sciences, University of Glasgow, Glasgow, United Kingdom; 2 Institute of Molecular Cell and Systems Biology, College of Medical, Veterinary and Life Sciences, University of Glasgow, Glasgow, United Kingdom; Emory University School of Medicine, UNITED STATES

## Abstract

Respiratory tract infections are of significant concern in the agriculture industry. There is a requirement for the development of well-characterised *in vitro* epithelial cell culture models in order to dissect the diverse molecular interactions occurring at the host-pathogen interface in airway epithelia. We have analysed key factors that influence growth and differentiation of ovine tracheal epithelial cells in an air-liquid interface (ALI) culture system. Cellular differentiation was assessed at 21 days post-ALI, a time-point which we have previously shown to be sufficient for differentiation in standard growth conditions. We identified a dose-dependent response to epidermal growth factor (EGF) in terms of both epithelial thickening and ciliation levels. Maximal ciliation levels were observed with 25 ng ml^-1^ EGF. We identified a strict requirement for retinoic acid (RA) in epithelial differentiation as RA exclusion resulted in the formation of a stratified squamous epithelium, devoid of cilia. The pore-density of the growth substrate also had an influence on differentiation as high pore-density inserts yielded higher levels of ciliation and more uniform cell layers than low pore-density inserts. Differentiation was also improved by culturing the cells in an atmosphere of sub-ambient oxygen concentration. We compared two submerged growth media and observed differences in the rate of proliferation/expansion, barrier formation and also in terminal differentiation. Taken together, these results indicate important differences between the response of ovine tracheal epithelial cells and other previously described airway epithelial models, to a variety of environmental conditions. These data also indicate that the phenotype of ovine tracheal epithelial cells can be tailored *in vitro* by precise modulation of growth conditions, thereby yielding a customisable, potential infection model.

## Introduction

Air is conducted into the lungs of mammals via the respiratory tract. The anatomical organisation and physiological function of the airway is such that it is constantly exposed to the atmosphere and hence represents a primary interaction site with bacteria, viruses and pollutants in the environment [1–3]. The epithelium lining the lumen of the airway possesses a complex cellular architecture with diverse cell types operating in concert to maintain lung and airway homeostasis. This is facilitated by providing an epithelial barrier that actively eliminates particulates, sensing environmental cues and regenerating damaged tissues [4,5]. In the trachea, these diverse functions are imparted by mucus-producing goblet cells, actively-beating ciliated cells, sensory brush cells and basal stem cells [6–9].

*In vitro* submerged tracheal epithelial cell cultures poorly reflect the complex cellular organisation associated with the airway epithelium [10,11]. However, by expanding to confluency on a semi-permeable membrane and culturing in specific media at an air-liquid interface (ALI), a more representative model of the *in vivo* tissue can be produced [12–15]. Models of the mouse, rat, guinea pig, cow, horse, sheep and human respiratory epithelia have been produced *in vitro* with varying degrees of differentiation being observed [12,16–22]. The extent to which *in vitro* primary airway cultures differentiate and reflect the *in vivo* tissue is dependent upon a wide variety of growth parameters including growth substrate properties, atmospheric gas composition, growth factor concentrations, culture period and passage number [23,24]. Importantly, while many of these factors have been analysed in detail for human tissues, *in vitro* animal systems remain poorly understood.

Since the development of the biphasic chamber-based culture system, which allows for ALI growth, there have been extensive efforts to optimise the conditions for differentiation of human airway epithelia. During early attempts to develop a defined growth medium for human tracheobronchial epithelia at ALI, the importance of carefully optimising the concentrations of epidermal growth factor (EGF), retinoic acid (RA) and triiodothyronine (T3) was greatly appreciated [13,25]. These studies led to the formulation of a standardised medium with fixed concentrations of these key growth factors known as Gray’s medium [13]. While this medium and subtle variations are still used for the culture of human airway epithelia, the levels of differentiation of airway epithelial cells from other species has been widely variable. Indeed, reported concentrations of EGF used in animal models vary from 0.5 ng/ml to 25 ng/ml [26,27] and concentrations of RA from 10 nM to 100 nM have been used [28,29]. Some studies exclude T3 at ALI [17,26,28], while others include a concentration of 6.5 ng/ml (10 nM) [29,30]. Furthermore, serum-containing media [20,29] or artificial serum-substitutes with proprietary formulations such as Ultroser G [31,32] or NuSerum [27] rather than defined concentrations of specific growth factors have also been used. Thus, it is clear that further research is required to gain an improved understanding of how airway epithelia from diverse species respond to these key growth factors.

In the agriculture industry, respiratory infections cause significant economic losses in the form of treatment expenses and mortality of livestock. Infections by bacterial pathogens such as *Mannheimia haemolytica*, *Pasteurella multocida*, *Histophilus somni* and *Mycoplasma ovipneumoniae* are of particular concern in the sheep-farming industry [33–36]. Many advances have been made in the study of human respiratory pathologies, in part due to extensive efforts in the development of highly representative *in vitro* models [37]. Such well-optimised and well-developed models are lacking in the area of veterinary respiratory research. Historically, costly and ethically questionable *in vivo* animal experimentation was employed to dissect the mechanisms involved in respiratory pathologies. There is clearly a need to employ more ethical, cost-effective and high-throughput means of studying infections that are relevant to the agriculture industry. Differentiated *in vitro* airway epithelial cell cultures grown at ALI represent significant advancements in these key areas and will enable the study of colonisation and virulence-related properties of respiratory pathogens at a molecular level.

We recently presented an analysis of the differentiation of ovine tracheal epithelial cells over time under standard ALI growth conditions [21]. We sought to expand on this knowledge by comparing a variety of important growth parameters and assessing the differentiation of the model at 21 days post-ALI. The results of the present study will allow for modification of a variety of important features of the *in vitro* ovine tracheal epithelial cell culture model and have important implications for model optimisation. Critically, there appear to be marked species-specific differences in the reported optimum concentrations of growth factors and other growth conditions and the results of the present study will be discussed in the context of the available literature.

## Materials and methods

### Ovine tracheal epithelial cell harvest and expansion

Tracheal specimens from freshly-slaughtered sheep were obtained from a local abattoir (Sandyford Abattoir, Paisley, UK) and were placed in ice-cold transport medium (PBS, 1% [v/v] penicillin-streptomycin [Pen-Strep] and 1% [v/v] Fungizone). Tracheae were longitudinally dissected to reveal the inner epithelium. Small pieces (~0.5 cm^2^) of *ex vivo* trachea were dissected and fixed in 2% (w/v) formaldehyde for histological assessment. Epithelia were carefully peeled from the underlying cartilage and connective tissue, placed in ice-cold digestion medium (Dulbecco’s minimal Eagle’s medium [DMEM], 10 μg ml^-1^ DNase, 1 mg ml^-1^ dithiothreitol and 1 mg ml^-1^ protease XIV from *Streptomyces griseus* [Sigma-Aldrich]), and digested at 4°C overnight. Digestion was halted by the addition of foetal calf serum (FCS) to a final concentration of 10% (v/v) and epithelial cells were removed by vigorous washing. The resultant cell suspension was strained through a 70 μm strainer, centrifuged at 300 × *g* for 5 min and washed with serum-containing growth medium (SGM), which comprised a 1:1 mixture of DMEM/Ham’s F12 with 10% (v/v) FCS, 1% (v/v) Pen-Strep and 1% (v/v) Fungizone. Cell pellets were resuspended in either SGM or airway epithelial growth medium (AEGM [Promocell, #C-21160]) containing 1% [v/v] Pen-Strep and 1% [v/v] Fungizone and seeded into T75 cell culture flasks. Flasks were routinely incubated at 37°C in 5% CO_2_ and 14% O_2_.

### Generation of differentiated ALI cultures

When flask cultures of epithelial cells reached ~70% confluency (4–6 days), the cells were removed by trypsinisation, washed and seeded onto Greiner 0.4 μm pore-diameter Thincerts of 1.0 × 10^8^ (#665640) or 2.0 × 10^6^ (#665641) pores cm^-2^ in SGM or AEGM at a density of 2.5 × 10^5^ cells per insert. Inserts were cultured under submerged conditions at 37°C in 5% CO_2_ and 7, 14 or 21% O_2_ until confluency was reached and a trans-epithelial electrical resistance (TEER) of at least 200 Ω × cm^2^ was achieved (4–14 days). At this point, the epithelial barrier was established and the cells were washed with PBS and fed with ALI medium (DMEM/AEGM base medium [1:1] supplemented with the following growth factors: 10 ng ml^-1^ epidermal growth factor [EGF], 100 nM retinoic acid [RA], 6.7 ng ml^-1^ triiodothyronine [T3], 5 μg ml^-1^ insulin, 500 ng ml^-1^ hydrocortisone, 500 ng ml^-1^ epinephrine and 10 μg ml^-1^ transferrin). From this point onwards, the ALI was maintained by feeding from the basal compartment only and exposing the apical surface to the atmosphere. Concentrations of EGF, RA and T3 in the ALI medium were varied as indicated in the results section. The growth phases in which these conditions were altered are highlighted in S1 Fig. The cells were gradually transitioned to ALI medium by feeding apically and basally with a 1:1 mix of SGM/ALI medium or AEGM/ALI medium approximately half-way through the submerged growth phase, prior to establishment of the ALI. Feeding and washing were carried out every 2–3 days throughout submerged and ALI growth. The ovine tracheal epithelial cells were cultured at ALI for 21 days before fixation and phenotypic analysis of cell layer properties.

### Immunofluorescence microscopy

At 21 days post-ALI, samples were washed with PBS and fixed by adding 0.5 ml 4% paraformaldehyde to the apical chamber and incubating for 15 min. All incubations were carried out at room temperature. The cells were washed with PBS and permeabilised with 0.5 ml permeabilisation buffer (PBS containing 0.5% [v/v] Triton X-100, 100 mg ml^-1^ sucrose, 4.8 mg ml^-1^ HEPES, 2.9 mg ml^-1^ NaCl and 600 μg ml^-1^ MgCl_2_, pH 7.2) for 10 min. The cells were washed three times with PBST (PBS containing 0.05% [v/v] Tween-20) and 0.5 ml blocking buffer (PBST containing 10% normal goat serum and 1% [w/v] BSA) was added to the apical surface and incubated for 1 h. The cells were washed three times with PBST and 0.1 ml primary antibody (rabbit anti-β-tubulin diluted 1 in 200 [Abcam, #ab6046] and mouse anti-ZO-1 diluted 1 in 50 [Thermofisher, #33–9100]), in blocking buffer, was added to the apical surface. After 1 h incubation at room temperature, the cells were washed three times and 0.1 ml secondary antibody (goat anti-rabbit Alexa Fluor 488 [Thermofisher, #A-11034] and goat anti-mouse Alexa Fluor 488 [Thermofisher, #A-11001]), diluted 1 in 400 in blocking buffer, was added. After 1 h incubation at room temperature, unbound antibody was removed by washing three times and the cells were stained with 0.1 ml rhodamine phalloidin (1U per sample) and 0.1 ml 300 nM DAPI for 20 min. The cells were washed three times with PBS, the membranes were cut from the inserts and placed cell-side up on glass slides. Five microlitres of Vectashield mountant were placed on the surface of the tissue, and a coverslip was carefully lowered onto the mountant and sealed in place using clear nail polish. Samples were analysed using a Leica Dmi 8 microscope. For quantitation of ciliation levels, five images were taken in different predetermined regions (i.e. top, bottom, centre, centre-left and centre-right) from each insert. A fluorescence intensity threshold was applied using ImageJ such that only high intensity cilial β-tubulin staining was above the threshold. The area above this threshold (AAT) was expressed as a percentage of the entire field area. Three inserts were analysed from each of four animals.

### Scanning electron microscopy

Following removal of basal medium, samples were fixed by adding 0.5 ml 1.5% (v/v) glutaraldehyde in 0.1 M sodium cacodylate to the apical surface and incubating at 4°C for 1 h. All subsequent incubations were carried out at room temperature. The cell layers were washed three times apically and basally with 0.1 M sodium cacodylate, and 0.5 ml 2% (w/v) osmium tetraoxide was added to each insert and incubated for 1 h. The cells were washed three times with distilled water and incubated for 1 h in 0.5% (w/v) uranyl acetate in the dark. A further two washes with distilled water were carried out before dehydrating the tissues via a series of increasing ethanol concentrations. The cell layers were finally incubated in hexamethyldisilizane for 1 h before drying in a desiccator overnight. The membranes were removed from the inserts and mounted cell-side up on aluminium SEM stubs using carbon tape. The stubs were sputter-coated with gold and samples were visualised using a Jeol 6400 electron microscope.

### Histological and immunohistochemical processing and analysis

Tracheal epithelial cell cultures were fixed with 0.5 ml 4% (w/v) paraformaldehyde for 15 min. Samples were washed with PBS and dehydrated via a series of increasing ethanol concentrations. The tissues were cleared with xylene and infiltrated with paraffin wax before embedding in wax blocks. Sections of 2.5 μm thickness were cut using a Thermoshandon Finesse ME+ microtome, followed by staining with haematoxylin and eosin (H&E) or periodic acid-Schiff’s (PAS) stain using standard histological procedures. Alternatively, sections were subjected to immunohistochemical labelling by processing with a Menarini Antigen Access Unit. After antigen retrieval, endogenous peroxide was blocked using H_2_O_2_ in PBS before incubating in mouse anti-p63 antibody (Abcam, #ab735) at a 1 in 30 dilution for 30 min. Primary antibody was labelled using an anti-mouse-HRP-labelled polymer and detected using a REAL EnVision Peroxidase/DAB+ Detection System (Dako, #K3468) according to the manufacturer’s instructions. Sections were counter-stained using Gill’s haematoxylin and dehydrated, cleared and mounted in synthetic resin. Visualisation was carried out using a Leica DM2000 microscope.

### Statistical analysis

Data were analysed using GraphPad Prism. Comparisons of each test with untreated/control samples were conducted using two-tailed Student’s *t*-test or one-way ANOVA with Dunnet’s post-test. Significance values are indicated by one (*P*<0.05), two (*P*<0.01) or three (*P*<0.001) asterisks.

## Results

### Epidermal growth factor (EGF) promotes ciliation and cell layer thickening

**Fig 1:**
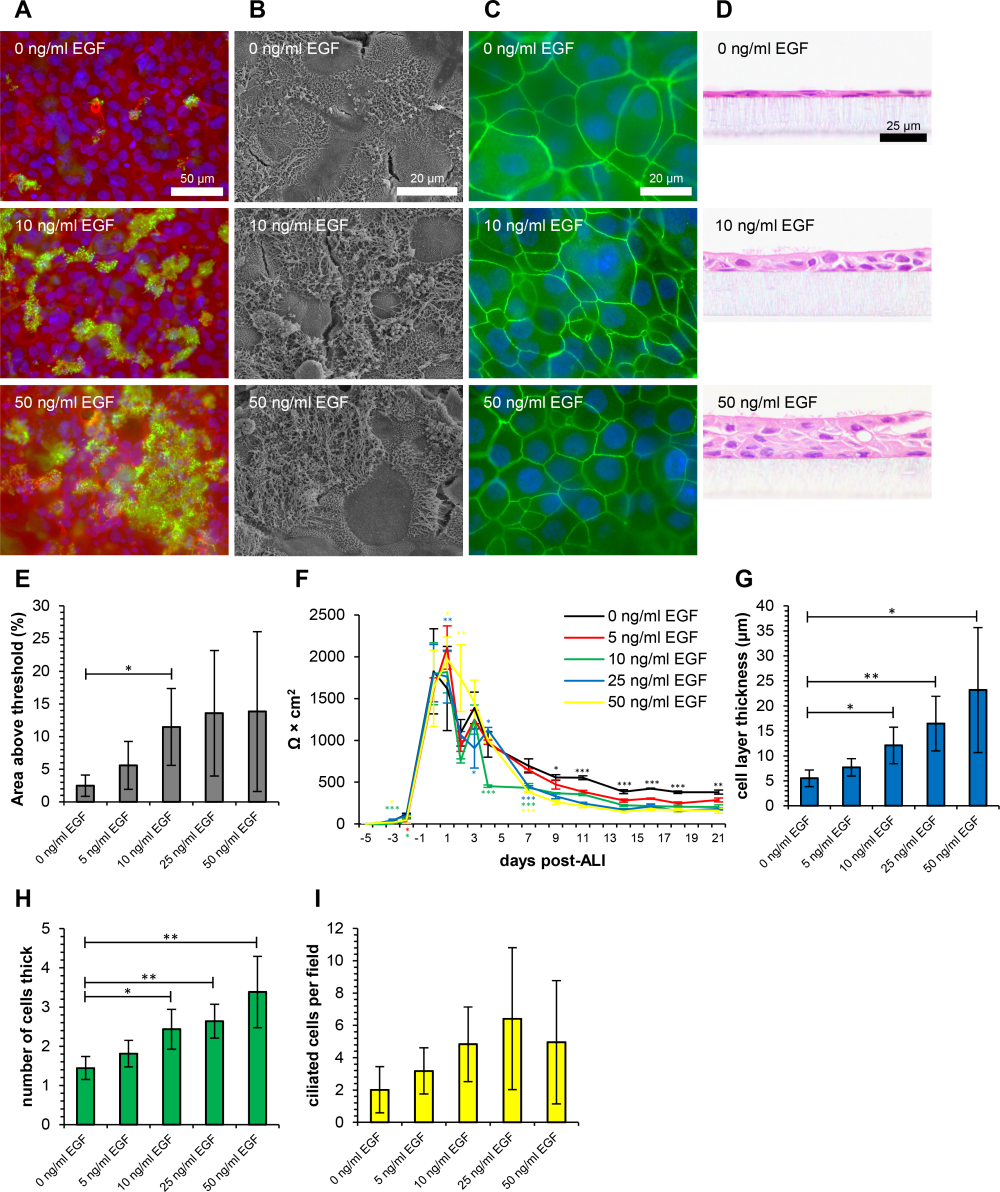
Epidermal growth factor plays diverse roles in the differentiation of ovine tracheal epithelial cells. Ovine tracheal epithelial cells were cultured at ALI for 21 days with the indicated concentrations of EGF. (A) Immunofluorescent staining with anti-β-tubulin (green), rhodamine-phalloidin (red) and DAPI (blue). (B) Scanning electron microscopy. (C) Immunofluorescent staining with anti-ZO-1 (green) and DAPI (blue). (D) Haematoxylin and eosin-stained histological sections. (E) Quantitation of ciliation as percentage of total area from β-tubulin staining. (F) Trans-epithelial electrical resistance measurements. Data shown are from a single representative animal with mean +/- standard deviation from three inserts displayed. (G) Cell layer thickness measured from three points per field in H&E-stained sections. (H) Cell layer thickness as determined by counting nuclei at three points per field in H&E-stained sections. (I) Quantitation of ciliation by counting ciliated cells in H&E-stained sections. (E, G, H, I) Five images from each of three inserts were analysed and data displayed is mean +/- standard deviation from four animals. Statistical significance was assessed by Student’s *t*-test (E, G, H and I) or one-way ANOVA with Dunnet’s post-test (F). Significance values are indicated by one (*P*<0.05), two (*P*<0.01) or three (*P*<0.001) asterisks. Black asterisks indicate all samples were significantly different to untreated control in panel F.

Differentiation of ovine airway epithelial cells was qualitatively assessed using immunofluorescent labelling of β-tubulin (Fig 1A), SEM (Fig 1B), immunofluorescent staining of the tight junction protein ZO-1 (Fig 1C) and histological analysis (Fig 1D). Quantitative assessment was performed by measuring the ciliated area above the fluorescence intensity threshold (i.e. Area Above Threshold: AAT) (Fig 1E), measuring TEER at the indicated time-points (Fig 1F), measuring the cell layer thickness in microns (Fig 1G), assessing the thickness of the cell layer in numbers of cells (Fig 1H) and counting the numbers of ciliated cells per field (Fig 1I) from H&E-stained histological sections. Additional methods used for analysis are included in S2 and S3 Figs. PAS staining for mucin-containing cells (S3B Fig) and immuno-labelling of the p63 basal stem cell transcription factor (S3C Fig) were also carried out. Additional counts for goblet cells (S3D Fig), vacuolated cells (S3E Fig) and pyknotic cells (S3F Fig) were also performed. For robustness, all analyses were carried out in four replicate animals. For assessment of variations in EGF concentration, a fixed concentration of 100 nM RA was used.

Ovine tracheal epithelial cells displayed a dose-dependent increase in abundance of ciliated cells with increasing EGF concentration up to 25 ng/ml (Fig 1A, 1B, 1D, 1E and 1I and S2A and S2B Fig). In the absence of EGF, only isolated patches of short cilia were observed while 25 and 50 ng/ml EGF resulted in dense regions of mature cilia, which were frequently coated with mucus globules. Although a large degree of variation was observed between animals, a dose-dependent increase in ciliation was observed up to 25 ng/ml using both immunofluorescence and histology-based assessment (Fig 1E and 1I). Maximal ciliation was determined as 13.8% AAT, which corresponds to 6.4 ciliated cells per field (Fig 1E and 1I). EGF also induced dose-dependent thickening of the cell layer (Fig 1G and 1H). This increase from 0 to 50 ng/ml was seen both in absolute thickness (5.5 to 23.2 μm) and numbers of cells (1.4 to 3.4 cells thick). As such, the increased thickness observed at higher EGF concentrations can largely be attributed to increasing numbers of cells, consistent with the mitogenic activity of this growth factor.

The junctional integrity of the cell layer was similar at all concentrations of EGF (Fig 1C) and the only observable difference in ZO-1 staining with increasing EGF concentrations was an apparent reduction in cell size and corresponding increase in the number of cells per field. All concentrations of EGF induced similar TEER profiles, although 0 ng/ml EGF resulted in a higher terminal TEER (381.3 Ω × cm^2^) than 50 ng/ml (159.5 Ω × cm^2^). Despite the drop in TEER from ~2000 Ω × cm^2^ to < 200 Ω × cm^2^, the barrier remained intact and no leakage of basal contents occurred. There were no apparent differences in PAS-positive or p63-positive cell numbers at different EGF concentrations (S3B and S3C Fig). While goblet cells were difficult to discern in the thin cell layers derived from 0 ng/ml EGF cultures, there were no clear dose-dependent increases in goblet cells with increasing EGF at the other concentrations tested (S3D Fig). While greater numbers of vacuolated cells were observed at 25 and 50 ng/ml EGF than lower concentrations, this is likely reflective of the increased cell numbers and cell layer thickness obtained at these concentrations (S3E Fig). Similarly, no clear incremental response was observed with EGF in terms of pyknotic cell death (S3F Fig).

### Retinoic acid (RA) is required for differentiation

**Fig 2:**
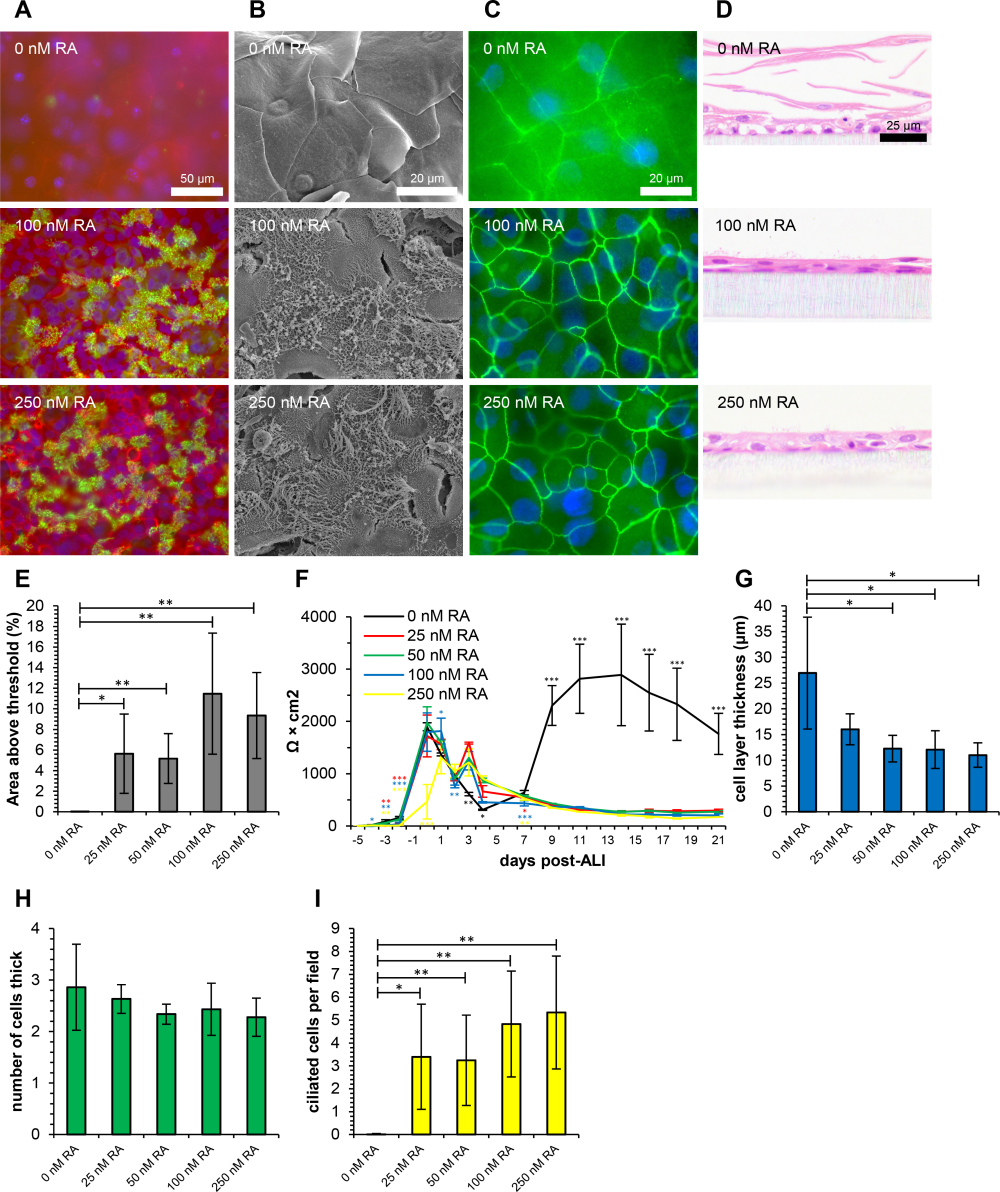
Retinoic acid is required for *in vitro* differentiation of ovine tracheal epithelial cells. Ovine tracheal epithelial cells were cultured at ALI for 21 days with the indicated concentrations of retinoic acid. (A) Immunofluorescent staining with anti-β-tubulin (green), rhodamine-phalloidin (red) and DAPI (blue). (B) Scanning electron microscopy. (C) Immunofluorescent staining with anti-ZO-1 (green) and DAPI (blue). (D) Haematoxylin and eosin-stained histological sections. (E) Quantitation of ciliation as percentage of total area from β-tubulin staining. (F) Trans-epithelial electrical resistance measurements. Data shown are from a single representative animal with mean +/- standard deviation from three inserts displayed. (G) Cell layer thickness measured from three points per field in H&E-stained sections. (H) Cell layer thickness as determined by counting nuclei at three points per field in H&E-stained sections. (I) Quantitation of ciliation by counting ciliated cells in H&E-stained sections. (E, G, H, I) Five images from each of three inserts were analysed and data displayed is mean +/- standard deviation from four animals. Statistical significance was assessed by Student’s *t*-test (E, G, H and I) or one-way ANOVA with Dunnet’s post-test (F). Significance values are indicated by one (*P*<0.05), two (*P*<0.01) or three (*P*<0.001) asterisks. Black asterisks indicate all samples were significantly different to untreated control in panel F.

Variation in the principle phenotypes of differentiation of ovine tracheal epithelial cells in response to RA were assessed qualitatively (Fig 2A–2D) and quantitatively (Fig 2E–2I) as previously described for EGF. The complete range of concentrations tested, together with PAS staining, p63 immunohistochemistry and additional quantitative data from histology are displayed in the supplementary information (S4 and S5 Figs). For assessment of variations in RA concentration, a fixed concentration of 10 ng/ml EGF was used.

In the absence of RA, the cultured airway epithelial cells formed a squamous, stratified cell layer completely devoid of cilia (Fig 2A–2D). Under these conditions, no specific labelling of cilial β-tubulin was observed by immunofluorescence (Fig 2A). The unusual phenotype of these RA-starved cells is particularly apparent in the SEM analysis. Here the cells appear extremely flattened, so much so that the nuclei are evident protruding from the centre of each sheet-like cell (Fig 2B). Overlapping sheets of cells with some peripheral ZO-1 staining are also apparent in immunofluorescence analysis; however, these junctions were not well defined and were atypical in appearance compared to normal tight junctions (Fig 2C). Sheets of squamous epithelial cells that have become separated from the underlying basal cells were also apparent in histological sections (Fig 2D and S5A–S5C Fig). The RA-deficient cultures also displayed an unusual TEER profile, with a secondary increase in TEER being observed from day 7, peaking at 2892 Ω × cm^2^ at day 13 (Fig 2F). Interestingly, this phase of growth correlates with the period at which differentiation normally begins to occur in ovine tracheal ALI cultures [21].

While culture in the absence of RA resulted in a dramatic undifferentiated phenotype, the range of concentrations tested (25 nM to 250 nM) resulted in little variation in differentiation. A dose-dependent decrease in absolute thickness was observed with increasing RA concentration from 27.0 μm at 0 nM to 11.0 μm at 250 nM RA (Fig 2G). A slight increase in ciliation was observed from 50 nM to 100 nM RA both by immunofluorescence microscopy (5.2% AAT to 11.5% AAT) (Fig 2E) and counting of ciliated cells in images of histological sections (3.3 to 4.8 cells per field) (Fig 2I). No differences were observed in the numbers of PAS-positive, p63-positive, goblet, vacuolated or pyknotic cells when comparing 25 to 250 nM RA (S5B–S5F Fig).

### Ovine tracheal epithelial cells display improved differentiation on high pore-density cell culture inserts

**Fig 3:**
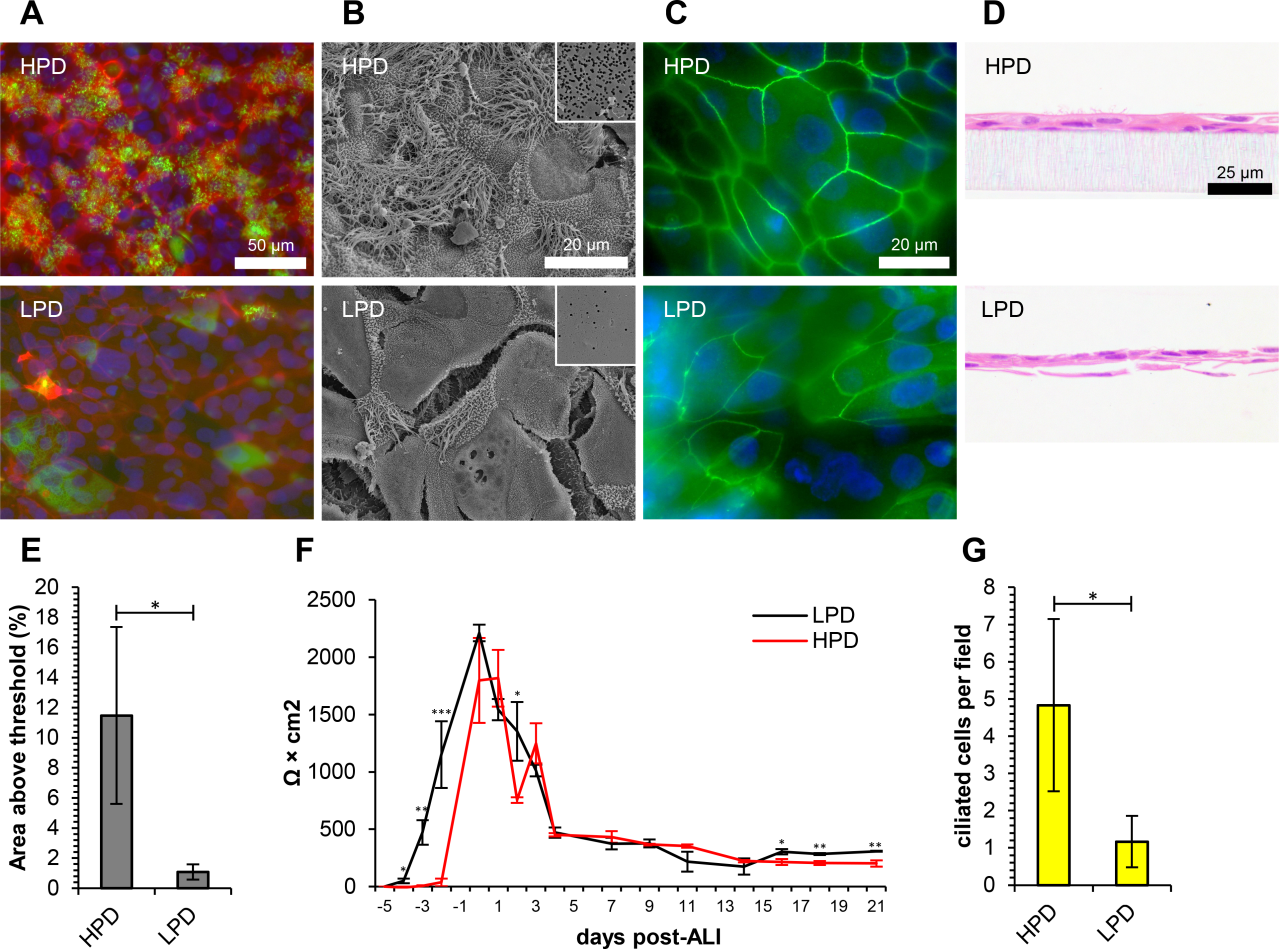
Pore-density of the culture membrane plays an important role in the differentiation of ovine tracheal epithelial cells. Ovine tracheal epithelial cells were cultured at ALI for 21 days on high pore-density (HPD) or low pore-density (LPD) cell culture inserts. (A) Immunofluorescent staining with anti-β-tubulin (green), rhodamine-phalloidin (red) and DAPI (blue). (B) Scanning electron microscopy, density of pores indicated inset. (C) Immunofluorescent staining with anti-ZO-1 (green) and DAPI (blue). (D) Haematoxylin and eosin-stained histological sections. (E) Quantitation of ciliation as percentage of total area from β-tubulin staining. (F) Trans-epithelial electrical resistance measurements. Data shown are from a single representative animal with mean +/- standard deviation from three inserts displayed. (G) Quantitation of ciliation by counting ciliated cells in H&E-stained sections. (E, G) Five images from each of three inserts were analysed and data displayed is mean +/- standard deviation from four animals. Statistical significance was assessed by Student’s *t*-test (E, F and G). Significance values are indicated by one (*P*<0.05), two (*P*<0.01) or three (*P*<0.001) asterisks.

Polyethylene terephthalate (PET) cell culture inserts are commonly available in two pore-densities, 2.0 × 10^6^ pores cm^-2^ (low pore-density [LPD]) and 1.0 × 10^8^ pores cm^-2^ (high pore-density [HPD]). The phenotypes of ovine tracheal epithelial cells when cultured for 21 days at ALI on LPD and HPD inserts were compared by β-tubulin immunostaining (Fig 3A), SEM (Fig 3B), ZO-1 immunostaining (Fig 3C), H&E staining of histological sections (Fig 3D), PAS staining (S6A Fig) and p63 immunohistochemistry (S6B Fig). Quantitative data from immunofluoresecence (Fig 3E), TEER measurement (Fig 3F) and histology (Fig 3G and S6C–S6G Fig) are also displayed.

The most striking difference between the two membranes tested was a dramatic increase in ciliation with HPD membranes compared with LPD membranes (Fig 3A, 3B, 3E and 3G). In comparison to HPD inserts, LPD inserts exhibited sparsely ciliated cells (Fig 3A and 3B) and β-tubulin staining was largely confined to cytoskeletal regions (Fig 3A), consistent with that of proliferating cells in the first few days post-seeding on HPD membranes [21]. HPD inserts displayed ciliation of 11.5% AAT with 4.8 ciliated cells per field compared with 1.1% AAT and 1.2 ciliated cells per field on LPD inserts (Fig 3E and 3G, respectively). Interestingly, epithelial barrier formation and a concurrent increase in TEER were observed two days earlier with LPD inserts (Fig 3F). Cell layers that were grown on LPD inserts appeared fragmented with inconsistent tight junctions and irregular cell morphology (Fig 3B–3D). Histological sections of LPD membrane-grown cultures typically contained many thin and squamous or fragmented areas and as such, the cell types depicted in the supplementary quantitative analysis in S6E–S6G Fig were difficult to discern and are therefore represented in lower numbers than in the HPD cultures.

### Ovine tracheal epithelial cells display enhanced ciliation when cultured at sub-ambient oxygen tension

**Fig 4:**
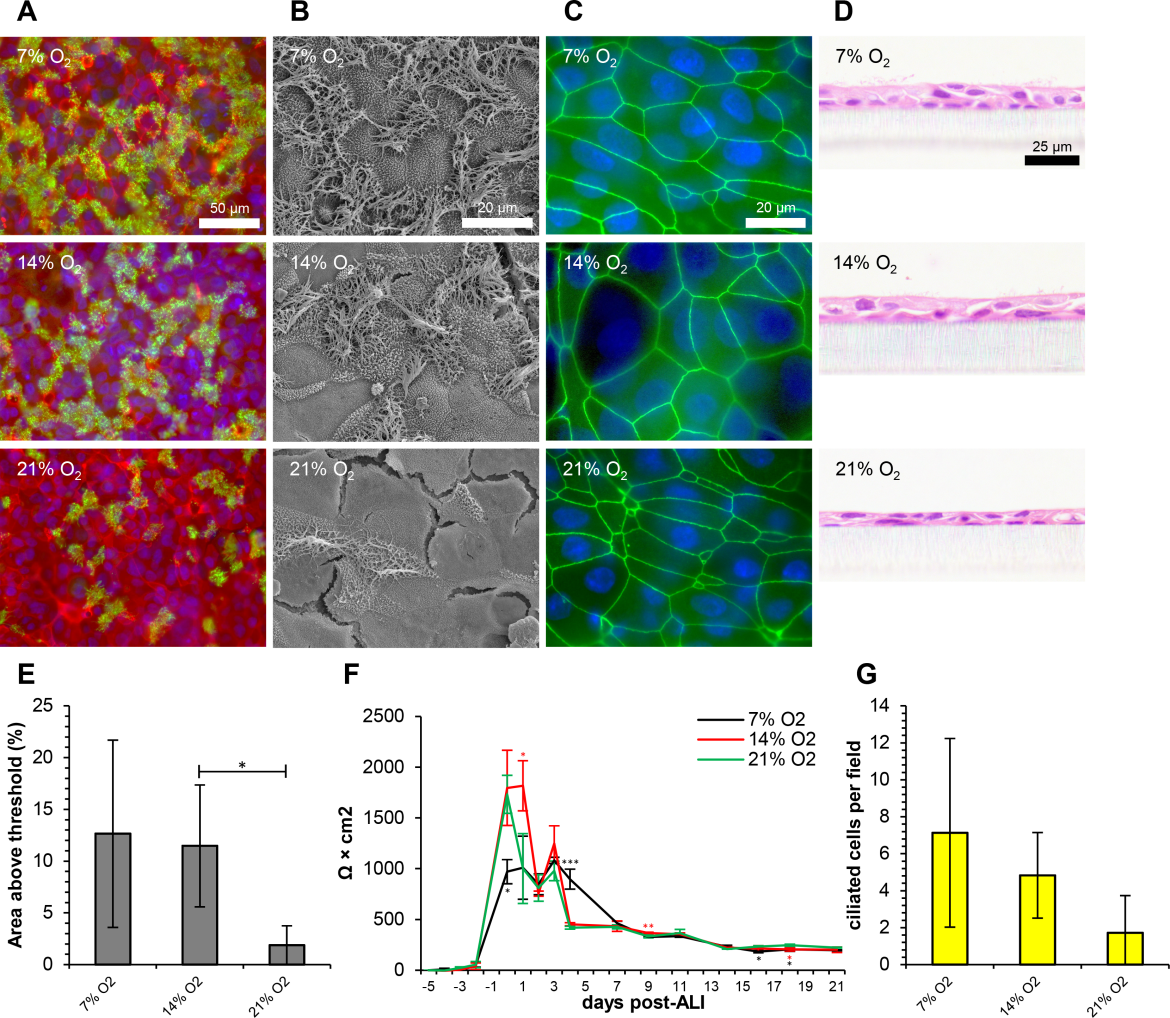
Ovine tracheal epithelial cells differentiate optimally at sub-ambient oxygen tension. Ovine tracheal epithelial cells were cultured in an atmosphere of 7, 14 or 21% O_2_. (A) Immunofluorescent staining with anti-β-tubulin (green), rhodamine-phalloidin (red) and DAPI (blue). (B) Scanning electron microscopy. (C) Immunofluorescent staining with anti-ZO-1 (green) and DAPI (blue). (D) Haematoxylin and eosin-stained histological sections. (E) Quantitation of ciliation as percentage of total area from β-tubulin staining. (F) Trans-epithelial electrical resistance measurements. Data shown are from a single representative animal with mean +/- standard deviation from three inserts displayed. (G) Quantitation of ciliation by counting ciliated cells in H&E-stained sections. (E, G) Five images from each of three inserts were analysed and data displayed is mean +/- standard deviation from four animals. Statistical significance was assessed by Student’s *t*-test (E and G) or one-way ANOVA with Dunnet’s post-test (F). Significance values are indicated by one (*P*<0.05), two (*P*<0.01) or three (*P*<0.001) asterisks.

The phenotypes of ovine tracheal cells cultured at 7, 14 and 21% O_2_ were compared microscopically (Fig 4A–4D) and quantitatively (Fig 4E–4G). As with all data presented thus far, AEGM growth medium was used during submerged growth conditions. The effect of modifying oxygen tension was also compared with serum-containing growth medium (SGM) at 7, 14 and 21% O2. Complete data for β-tubulin immunostaining, SEM, ZO-1 immunostaining, quantitation of ciliation and TEER are represented in S7A–S7E Fig. Complete histological data are represented in S8A–S8C Fig and complete quantitative data are presented in S8D–S8I Fig. A detailed discussion on comparing these submerged growth media will follow this section.

Culture at sub-ambient (7 and 14% O_2_) oxygen tension consistently resulted in improved ciliation compared with normal atmospheric oxygen tension. At 7 and 14% O_2_, large, dense patches of cilia were observed on the apical surface, while only isolated ciliated cells were observed at 21% O_2_ (Fig 4A and 4B). Tight junction integrity and architecture were similar across the range of oxygen concentrations tested (Fig 4C). The morphology of the cell layers also appeared similar in histological sections, with the exception of increased numbers of ciliated cells at 7 and 14% O_2_ (Fig 4D). Quantitatively, ciliation decreased from 12.6% AAT and 7.1 ciliated cells per field at 7% O_2_ to 1.9% AAT and 1.7 ciliated cells per field at 21% O_2_ (Fig 4E and 4G, respectively). Importantly, the effect of decreasing oxygen tension on augmentation of ciliation was also observed when ovine tracheal epithelial cells were cultured in SGM prior to establishment of the ALI (S7A, S7B, S7D and S8F Figs), further substantiating the importance of regulating oxygen tension during culture of ovine airway epithelial cells *in vitro*. There was little difference in barrier formation in response to varying oxygen tension as reflected by TEER measurement, although culture at 7% O_2_ resulted in a decreased peak TEER reading (Fig 4F). This was observed with the use of both AEGM (Fig 4F) and SGM (S7E Fig) as the submerged growth medium.

Similar numbers of PAS-positive and p63-positive cells were observed at all three oxygen concentrations and little difference was observed in thickness of the cell layer (S8A–S8E Fig). A slight increase in the numbers of goblet cells per field was observed in cultures grown at 7 (1.7) and 14% O_2_ (1.5) compared with 21% O_2_ (0.8) (S8G Fig). Little difference was observed in the cell death markers vacuolation and pyknosis (S8H and S8I Fig).

### The choice of submerged growth medium plays an important role in ovine tracheal epithelial cell differentiation *in vitro*

**Fig 5:**
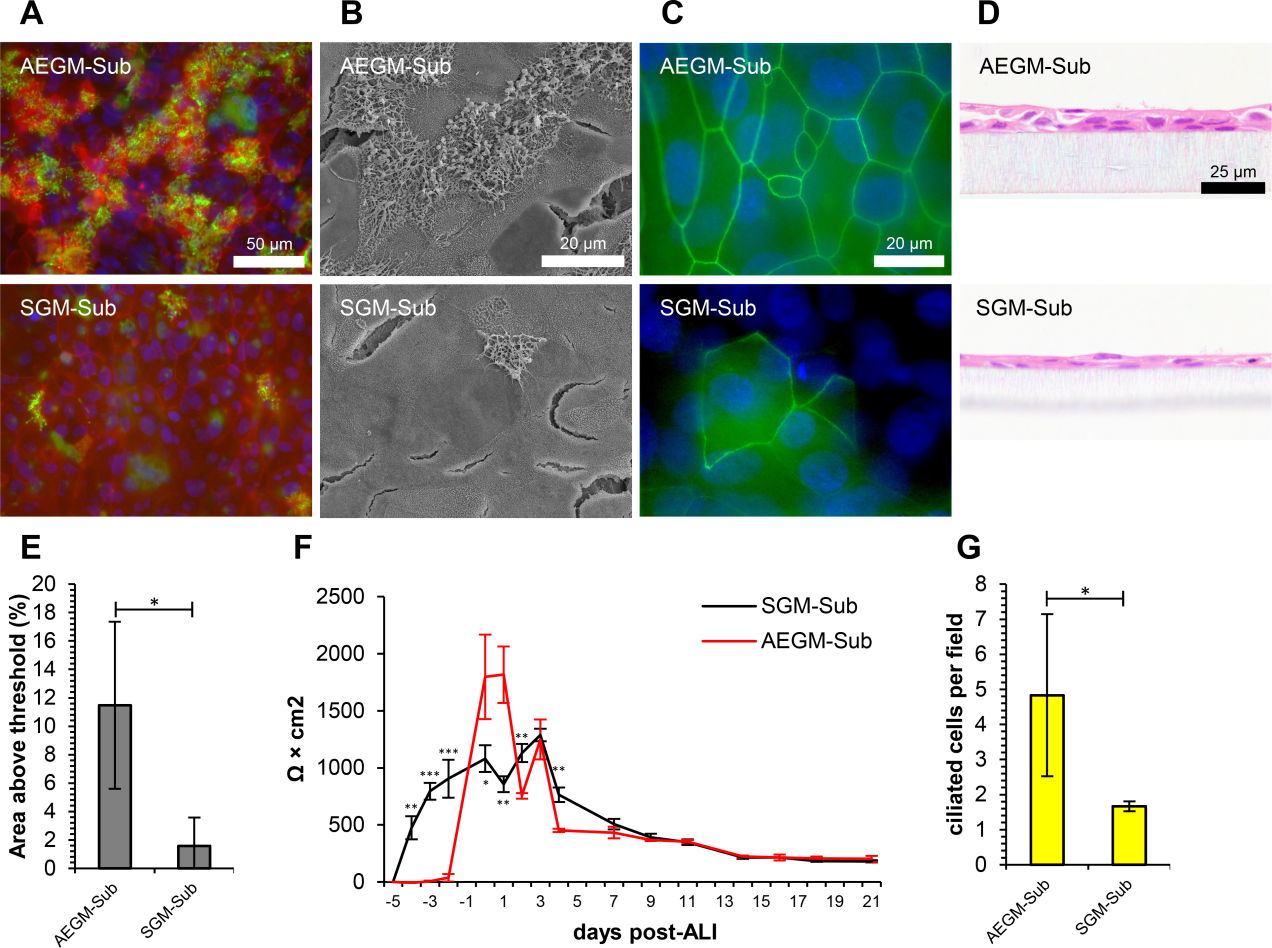
Submerged growth media have different effects on differentiation of ovine tracheal epithelial cells. Ovine tracheal epithelial cells were cultured to confluency in SGM or AEGM and at ALI for 21 days in ALI medium. (A) Immunofluorescent staining with anti-β-tubulin (green), rhodamine-phalloidin (red) and DAPI (blue). (B) Scanning electron microscopy. (C) Immunofluorescent staining with anti-ZO-1 (green) and DAPI (blue). (D) Haematoxylin and eosin-stained histological sections. (E) Quantitation of ciliation as percentage of total area from β-tubulin staining. (F) Trans-epithelial electrical resistance measurements. Data shown are from a single representative animal with mean +/- standard deviation from three inserts displayed. (G) Quantitation of ciliation by counting ciliated cells in H&E-stained sections. (E, G) Five images from each of three inserts were analysed and data displayed is mean +/- standard deviation from four animals. Statistical significance was assessed by Student’s *t*-test (E, F and G). Significance values are indicated by one (*P*<0.05), two (*P*<0.01) or three (*P*<0.001) asterisks.

During this study, we employed two different submerged media for both flask-based expansion of airway epithelial cells and for submerged growth on cell culture inserts. Despite the fact that the same medium (ALI medium) was used for the 21 days of culture at ALI, the choice of medium used for the 9 to 19-day period of submerged culture (comprising ~5 days of culture in flasks and 4–14 days of submerged culture on inserts before the epithelial barrier had formed) prior to this had a dramatic effect on the degree of differentiation observed. The cell layers were again compared using β-tubulin immunostaining (Fig 5A), SEM (Fig 5B), ZO-1 immunostaining (Fig 5C) and H&E staining of histological sections (Fig 5D). Quantitative data were obtained from β-tubulin immunostaining (Fig 5E), TEER measurements (Fig 5F) and from counting ciliated cells in histological sections (Fig 5G). Additional data derived from each medium at varying oxygen concentrations is indicated in S7 and S8 Figs.

Of all the conditions tested in this study, modification of submerged growth medium had the most significant impact on differentiation. The use of AEGM as a submerged growth medium allowed for the formation of a well-differentiated, highly ciliated epithelium with globules of mucus coating the cilia (Fig 5A and 5B). In comparison, the use of SGM during the submerged growth phase resulted in sparse, isolated patches of ciliation (Fig 5A and 5B). While intense and uniform ZO-1 staining was obtained with AEGM, staining was weaker and discontinuous in SGM-cultured cells (Fig 5C). The cell layers appeared thinner and had fewer ciliated cells when cultured in SGM during the submerged phase (Fig 5D and S8A Fig). Ciliation levels of 11.5% AAT and 4.8 ciliated cells per field were obtained when AEGM was employed, compared with 1.6% AAT and 1.7 ciliated cells per field with SGM (Fig 5E and 5G, respectively). Paradoxically, the ovine tracheal cells appeared to proliferate and form a barrier more rapidly with SGM as the TEER was found to increase to 474.6 Ω × cm^2^ one day after seeding, whereas 4 days were required for the cell layer to reach a TEER of 37.7 Ω × cm^2^ with AEGM as the submerged medium (Fig 5F). However, at the time of sampling (21 days post-ALI) the TEER was similar regardless of the submerged growth medium used (Fig 5F), a result that is somewhat surprising given the apparent poor tight junction integrity of cells when SGM was employed for submerged growth, as observed by immunostaining (Fig 5C). When comparing the use of AEGM with SGM at the three oxygen concentrations tested, it was apparent that AEGM promoted a thicker, more ciliated epithelium with improved tight junction integrity and more goblet cells (S7 and S8 Figs).

### Triiodothyronine (T3) does not affect differentiation of ovine tracheal epithelial cells

The complex growth medium formulation that is commonly used for the culture of airway epithelia from various species at ALI contains in addition to EGF, RA and other growth factors, T3, which has been reported to be important for mucociliary development and differentiation [38–40]. We cultured ovine tracheal epithelial cells at ALI either in the presence or absence of 6.7 ng ml^-1^ T3 and analysed differentiation using the procedures previously described. As displayed in S9 Fig, the cell layers displayed similar levels of ciliation, tight junction formation, epithelial thickness, PAS staining, p63 labelling, goblet cells, vacuolated cells, pyknotic cells and a similar pattern in TEER regardless of the inclusion or exclusion of T3. As such, it is apparent that T3 does not affect the differentiation characteristics of ovine tracheal epithelial cells which were tested in this study.

A summary of the results obtained through comparison of the growth conditions tested in this study is provided in Table 1.

**Table 1:** Summary of parameters tested, and degree of cellular differentiation observed.

Test	Specific concentrations/parameters tested (degree of differentiation observed)				
EGF	0 ng/ml (+)^2^	5 ng/ml (++)^3^	10 ng/ml (+++)^4^	25 ng/ml (+++)	50 ng/ml (+++)
RA	0 nM (-)^1^	25 nM (+++)	50 nM (+++)	100 nM (+++)	250 nM (+++)
Pore-density	Low (+)	High (+++)			
Oxygen tension	21% (+)	14% (+++)	7% (+++)		
Submerged growth medium	SGM (+)	AEGM (+++)			
T3	0 ng/ml (+++)	6.7 ng/ml (+++)			

^1^ (-) Cell layer squamous and stratified with no ciliated/mucus-producing cells

^2^ (+) cell layer squamous with very isolated patches of ciliated cells

^3^ (++) many ciliated and mucus-producing cells evident

^4^ (+++) extensive regions of pseudo-stratified highly ciliated epithelium, coated in mucus-like secretions.

## Discussion

In the present study, we have demonstrated that successful ovine tracheal epithelial cell differentiation at ALI is dependent upon a variety of environmental conditions. By comparing a comprehensive array of growth parameters, we have expanded upon previous knowledge of mammalian airway epithelial culture systems and facilitated the tailored development of a highly representative model for use in respiratory infection modelling.

Epidermal growth factor is a mitogen, which plays a central role in embryonic development and differentiation, a property that has been exploited to great effect in primary cell culture. Epidermal growth factor acts by binding to the EGF-receptor (EGFR) in host tissues and triggering auto-phosphorylation of receptor tyrosine kinases, which subsequently activate signal transduction cascades leading to cellular proliferation and differentiation [41]. EGFR-/- mice display thin and poorly developed airway epithelia [42], while *in utero* infusion of foetal lambs with EGF results in severe airway epithelial hyperplasia [43], consistent with the mitogenic activities of EGF. Administration of EGF results in proliferation of goblet cells *in vivo* in rats and causes increased expression of the major secretory mucin MUC5AC both *in vivo* and in cultured human airway epithelial cells *in vitro* [44].

The importance of EGF in the growth and differentiation of airway epithelia *in vitro* is therefore widely appreciated, but few studies have explored the effect of varying the concentration of this key mediator of differentiation on the observed phenotype of the ALI culture. When developing the standardised Gray’s medium for the culture of human tracheobronchial cells, it was found that EGF concentrations of 25 ng/ml that were previously commonly used [12,17,45], adversely affected growth, mucin production and morphology [13]. Accordingly, a concentration of 0.5 ng/ml was suggested as optimal [13]. When swine airway epithelial cells were cultured in Gray’s medium (0.5 ng/ml EGF), a thin, poorly differentiated epithelium with very few ciliated cells was developed [30]. A concentration of at least 1 ng/ml was required to induce ciliation, with little increase being observed with higher concentrations [30]. A dose-dependent increase in cell layer thickness was observed with a 2–3 cell thick epithelium being observed with 1 ng/ml EGF and an average of 8 cells thick being observed at 10 ng/ml with isolated regions of pronounced overgrowth/hyperplasia [30]. We recently demonstrated a dose-dependent increase in both cell layer thickness and ciliation in a bovine bronchial epithelial ALI model [46]. Optimal ciliation of 28 ciliated cells per field, with a similar thickness to the *in vivo* bronchial epithelium was observed at 10 ng/ml EGF [46]. A concentration of 10 ng/ml EGF has also been used for airway epithelial cell cultures from horses [20] and pigs [47], whereas up to 25 ng/ml has been used for ALI cultures from mice [16] and rats [17]. However, it has also been demonstrated that removal of EGF from the medium results in a four-fold increase in ciliated surface area of rat tracheal cell cultures [48]. In our study, 25–50 ng/ml EGF was required to induce maximal ciliation levels (Fig 1A, 1B, 1E and 1I). A dose-dependent increase in epithelial thickness was observed up to 50 ng/ml (23.2 μm, 3.4 cells thick), although the *in vitro* epithelium was still considerably thinner than the *in vivo* airway epithelium (mean thickness = 68.4 μm, S3 Fig). At this concentration, the epithelial cell layer was >3 cells thick and transitioning to a stratified morphology rather than the desired 2–3 cell thick pseudo-stratified epithelium. As such, a concentration of 25 ng/ml would allow for optimum ciliation and an appropriate compromise between increased epithelial thickness and retention of the pseudo-stratified morphology associated with this tissue type.

There have also been contrasting reports of EGFR activation either acting to increase expression of the major secretory mucin MUC5AC [44], or inhibit mucus cell differentiation *in vitro* [25]. In our study, we observed little difference in PAS staining or goblet cell differentiation across the four concentrations of EGF tested (S3B and S3D Fig). The marked differences in the responses of various species/tissue types to EGF highlight the importance of this type of comprehensive analysis and indicate that specific EGF concentrations should be tailored for each species.

Retinoic acid is an oxidised derivative of vitamin A, which is readily produced by retinol dehydrogenases and is responsible for a large proportion of the bioactivity of vitamin A [49]. The importance of RA in cellular differentiation and tissue development has long been appreciated. It was observed in 1925 that when rats were deprived of vitamin A, the normal tracheal epithelium became thickened, stratified and squamous consistent with squamous cell metaplasia [50]. A similar effect has been described when RA is removed from the culture medium of airway epithelial cells during ALI growth [13,17,30,38,51]. Interestingly, a similar thickened stratified squamous epithelium with decreased transcription of MUC5AC and concurrent decrease in mucin secretion was observed during deprivation of RA from middle ear epithelial cells *in vitro* [39]. While the majority of studies involving airway epithelial ALI cultures have used a concentration of 50 or 100 nM RA, well-differentiated canine (0 nM [52]), human (0.33 nM [11]), and mouse (10 nM [16,28]) airway epithelia have been developed with much lower concentrations. The work of Bateman *et al*. (2013) demonstrated a dose response of porcine airway epithelial cells to RA, with optimum ciliation being detected at the highest concentration tested (100 nM, 6.5 ciliated cells per field) [30]. A more recent study involving the culture of newborn pig tracheal epithelial cells employed a concentration of 0.33 nM RA, resulting in the formation of short immature cilia and concurrent diffuse β-tubulin immunostaining [47]. Our recent analysis of the response of bovine bronchial epithelial cells to RA also revealed a dose response with maximal ciliation at 100 nM RA [46]. While a clear dose response was not observed in ciliation of ovine tracheal epithelial cells, RA was required, and maximal levels were detected at 100/250 nM (11.5% AAT, 5.33 ciliated cells per field, Fig 2E and 2I) in reasonable agreement with other animal models.

Retinoic acid has also been described as an important inducer of secretory products from ALI cultures, particularly mucin [13,25,38,51,53]. While no mucus globules (Fig 2B), PAS-positive cells (S5B Fig) or cells with goblet cell-like appearance (S5D Fig) were detected in the absence of RA, little difference was observed comparing the different concentrations of RA tested. We observed a similar pattern with respect to goblet cell formation in the bovine airway epithelial model [46]. As such, it appears that RA is required for the differentiation of bovine and ovine airway mucus-producing goblet cells *in vitro* but a clear dose response is not observed.

A wide variety of microporous polymer membranes have been described for the culture of airway epithelial cells at ALI, including: polyester, polyethylene terephthalate (PET), hydrophilic polytetrafluoroethylene (PTFE), polycarbonate and mixed cellulose esters [54]. Ultrathin ceramic membranes have also been described [55]. While the issue of pore-density has not specifically been addressed in relation to airway epithelial cell differentiation, a number of other growth substrate properties including coating and material comparisons have been carried out. A large number of studies employ collagen-coated membranes. It has been reported that in the absence of type I collagen, ciliation is markedly reduced and mucus cell differentiation is delayed in a rat tracheal epithelial cell culture model [56]. Collagen can either be applied as a thin film or used as a thick collagen gel. It has been reported that a thick collagen gel (>1 mm thick) is required for optimal mucociliary differentiation [57]. We have demonstrated that mucociliary differentiation of airway epithelia from cattle [46] and sheep (Figs 1–5 and S2–S9 Figs) occurs in the absence of collagen. In our hands, collagen coating of membranes had no effect on differentiation but did result in more consistent attachment of cells and a more rapid increase in TEER (data not shown). Choice of membrane material can influence mucociliary differentiation and epithelial thickness with polytetrafluoroethylene (PTFE) membranes (undefined pore-density) yielding a thicker and more ciliated epithelium than PET (4.0 × 10^6^ pores cm^-2^) [58]. PET membranes with 0.4 μm pores are typically provided at a pore-density of 2.0 × 10^6^, 4.0 × 10^6^ or 1.0 × 10^8^ pores cm^-2^. Pore-density has been described to influence growth rates of Caco-2 and MDCK cells [59], but limited comparisons have been made with primary airway cultures. We observed poor levels of differentiation and inconsistent growth/cell health with LPD inserts (Fig 3 and S6 Fig). This was also observed when bovine bronchial cells were cultured on similar inserts [46]. It is possible that diffusion of medium is somewhat compromised at this lower pore-density. While pore-density of PTFE inserts is impossible to assess due to the fibrous structure of the membrane, it is possible that reports of improved differentiation with PTFE [58] and HPD PET [46] compared with LPD PET inserts are primarily due to improved diffusion, particularly where 0.4 μm pores are employed. This issue should be carefully considered when choosing an appropriate insert for each application.

Decreased oxygen tension (below ambient/atmospheric O_2_ = 21%) has been shown to be beneficial for the growth and differentiation of some primary cells/tissues. Mouse embryonic fibroblasts accumulated less DNA damage when cultured at 3% O_2_, thereby inhibiting senescence and extending the proliferative capacity of the cells compared with culture at 21% O_2_ [60]. Murine embryonic airway explants exhibited enhanced branching and morphogenesis at 3% O_2_ compared with 20% O_2_, while maintaining proper differentiation characteristics [61]. In the case of embryonic stem cells, exploitation of the full range of physiological oxygen tensions and step-wise changes in oxygen tension were described as powerful tools in the optimisation of cellular differentiation [62]. Given that airway basal cells possess a degree of multipotency [9] and the fact that the airway epithelium is exposed to a gradient of oxygen concentrations varying from 21% (inhaled atmospheric air) to 14–17% (exhaled air), we hypothesised that modulation of oxygen tension during culture may result in alterations in the differentiation status of the mature ALI culture. We recently described the effect of decreased oxygen tension on the differentiation of bovine bronchial epithelial cells [46]. In both this study and that of Cozens *et al*. (2017), a dose response was observed, with lower concentrations of O_2_ resulting in enhanced ciliated cell differentiation. It has been shown that culture of normal human bronchial epithelial cells under hypoxic (0.5% O_2_) conditions leads to inhibition of ciliation [63]. It is possible that concentrations of O_2_ lower than the 7% tested in this study would be similarly detrimental to ovine airway epithelial cells. To our knowledge, we are the first group to report a positive effect of decreased oxygen tension on primary airway epithelial cell differentiation at ALI.

Serum-containing growth medium (SGM) has been extensively applied for the initial expansion and proliferation phases of airway epithelial cell growth [20,29,31,46,64]. Enhanced attachment efficiency (~two-fold) when seeding inserts in SGM has been described for canine tracheal epithelial cells [52]. When seeded in this medium the cells were considerably larger and more squamous and if maintained in this medium at ALI, failed to develop an appropriate pseudo-stratified ciliated morphology [52]. We routinely use SGM for the submerged phases of bovine bronchial epithelial cell growth, and achieve excellent levels of mucociliary differentiation [46]. However, when ovine tracheal epithelial cells are cultured in SGM before transitioning to complete ALI medium and establishing the ALI they form a thinner cell layer with fewer ciliated and goblet cells compared with the use of AEGM (Fig 5 and S7 and S8 Figs). Thus, the cells attach, proliferate more rapidly and form a barrier more quickly with SGM (Fig 5F) but this comes at the expense of reduced downstream differentiation. In some experiments with AEGM, barrier formation and subsequent increase in ovine tracheal epithelial TEER required up to 14 days; however, this issue can be resolved by coating the growth substrate with type I collagen. Interestingly, when we attempted to culture bovine bronchial epithelial cells in AEGM, the cells exhibited poor cell attachment and growth, and thus failed to form a barrier for up to 14 days. This highlights an interesting species-specific difference in optimal culture conditions. While carefully calibrated conditions are typically employed during culture at ALI, the specific medium formulation prior to ALI establishment is a factor that is often overlooked. Here, we have demonstrated that growth conditions in this early phase of model development can have a significant impact on the level of differentiation observed.

In addition to EGF and RA, triiodothyronine (T3), a common component of airway epithelial growth media, has been reported to be involved in the regulation of *in vitro* mucociliary differentiation. Decreased goblet cell differentiation and repression of the secretory phenotype of ALI cultures, specifically via repression of the major secretory mucin MUC5AC, have been described [38–40]. In spite of these reports, T3 is included as a component of proprietary airway growth media and the majority of studies have included T3 during culture at ALI. In our bovine bronchial and ovine tracheal airway epithelial cell culture models, little difference was seen in the differentiation status of the epithelium in terms of ciliation, surface mucus production (viewed by SEM), PAS staining or goblet cell production ([46], S9 Fig) in the presence or absence of T3. As such, in our ruminant models at least, it appears that the inclusion or removal of T3 does not affect differentiation of the *in vitro* airway epithelium.

In summary, we have carried out the most extensive characterisation to date of growth conditions for the development of optimally differentiated ovine airway epithelial cell cultures. The results of this study will allow for greater consistency and robustness in the *in vitro* generation of highly representative airway tissues for application in the investigation of veterinary pathologies, particularly in the study of bacterial and/or viral pathogen interactions. We have also revealed the importance of various growth factors in modulating innate immune response phenotypes associated with the respiratory epithelium (e.g. ciliation, mucus production and barrier formation). This will allow for the dissection of the relative roles played by each of these key properties in relation to defence from invading pathogens.

## Supporting information

S1 Fig Medium formulation/conditions used at each phase of growth. The tests carried out in this study (A-F) are outlined together with the duration of each growth phase. Specific medium formulations are detailed. The parameters which were varied in each test are underlined.
(TIF)

S2 Fig Epidermal growth factor plays diverse roles in the differentiation of ovine tracheal epithelial cells. Ovine tracheal epithelial cells were cultured at ALI for 21 days with the indicated concentrations of EGF. (A) Immunofluorescent staining with anti-β-tubulin, rhodamine-phalloidin and DAPI. (B) Scanning electron microscopy. (C) Immunofluorescent staining with anti-ZO-1 and DAPI.
(TIF)

S3 Fig Epidermal growth factor plays diverse roles in the differentiation of ovine tracheal epithelial cells. Ovine tracheal epithelial cells were cultured at ALI for 21 days with the indicated concentrations of EGF. (A) Haematoxylin and eosin-stained histological sections. (B) Periodic acid-Schiff-stained histological sections. (C) Anti-p63 IHC of histological sections; p63-positive cells exhibit brown nuclei. (D) Number of goblet cells per field in H&E-stained sections. (E) Number of vacuolated cells per field in H&E-stained sections. (F) Number of cells exhibiting pyknotic nuclei in H&E-stained sections. (D-F) Five images from each of three inserts were analysed and data displayed is mean +/- standard deviation from four animals. Statistical significance was assessed by Student’s *t*-test (D-F). Significance values are indicated by one (*P*<0.05), two (*P*<0.01) or three (*P*<0.001) asterisks.
(TIF)

S4 Fig Retinoic acid is required for *in vitro* differentiation of ovine tracheal epithelial cells. Ovine tracheal epithelial cells were cultured at ALI for 21 days with the indicated concentrations of retinoic acid. (A) Immunofluorescent staining with anti-β-tubulin, rhodamine-phalloidin and DAPI. (B) Scanning electron microscopy. (C) Immunofluorescent staining with anti-ZO-1 and DAPI.
(TIF)

S5 Fig Retinoic acid is required for *in vitro* differentiation of ovine tracheal epithelial cells. Ovine tracheal epithelial cells were cultured at ALI for 21 days with the indicated concentrations of retinoic acid. (A) Haematoxylin and eosin-stained histological sections. (B) Periodic acid-Schiff-stained histological sections. (C) Anti-p63 IHC of histological sections; p63-positive cells exhibit brown nuclei. (D) Number of goblet cells per field in H&E-stained sections. (E) Number of vacuolated cells per field in H&E-stained sections. (F) Number of cells exhibiting pyknotic nuclei in H&E-stained sections. (D-F) Five images from each of three inserts were analysed and data displayed is mean +/- standard deviation from four animals. Statistical significance was assessed by Student’s *t*-test (D-F). No significant differences were observed.
(TIF)

S6 Fig Pore-density of the culture membrane plays an important role in the differentiation of ovine tracheal epithelial cells. Ovine tracheal epithelial cells were cultured at ALI for 21 days on high pore-density (HPD) or low pore-density (LPD) cell culture inserts. (A) Periodic acid-Schiff-stained histological sections. (B) Anti-p63 IHC of histological sections; p63-positive cells exhibit brown nuclei. (C) Cell layer thickness measured from three points per field in H&E-stained sections. (D) Cell layer thickness as determined by counting nuclei at three points per field in H&E-stained sections. (E) Number of goblet cells per field in H&E-stained sections. (F) Number of vacuolated cells per field in H&E-stained sections. (G) Number of cells exhibiting pyknotic nuclei in H&E-stained sections. (C-G) Five images from each of three inserts were analysed and data displayed is mean +/- standard deviation from four animals. Statistical significance was assessed by Student’s *t*-test (C-G). Significance values are indicated by one (*P*<0.05) or three (*P*<0.001) asterisks.
(TIF)

S7 Fig Ovine tracheal epithelial cells differentiate optimally at sub-ambient oxygen tension with AEGM as submerged growth medium. Ovine tracheal epithelial cells were cultured to confluency (while submerged) on cell culture inserts in SGM or AEGM and at ALI in ALI medium. A humidified atmosphere comprising 5% CO_2_ and either 7, 14 or 21% O_2_ was employed for submerged and ALI growth phases. (A) Immunofluorescent staining with anti-β-tubulin, rhodamine-phalloidin and DAPI. (B) Scanning electron microscopy. (C) Immunofluorescent staining with anti-ZO-1 and DAPI. (D) Quantitation of ciliation as percentage of total area from β-tubulin staining. Five images from each of three inserts were analysed and data displayed is mean +/- standard deviation from four animals. (E) Trans-epithelial electrical resistance measurement. Data shown are from a single representative animal with mean +/- standard deviation from three inserts displayed.
(TIF)

S8 Fig Ovine tracheal epithelial cells differentiate optimally at sub-ambient oxygen tension with AEGM as submerged growth medium. Ovine tracheal epithelial cells were cultured to confluency in SGM or AEGM and at ALI in ALI medium. A humidified atmosphere comprising 5% CO_2_ and either 7, 14 or 21% O_2_ was employed for submerged and ALI growth phases. (A) Haematoxylin and eosin-stained histological sections. (B) Periodic acid-Schiff-stained histological sections. (C) Anti-p63 IHC of histological sections; p63-positive cells exhibit brown nuclei. (D) Cell layer thickness measured from three points per field in H&E-stained sections. (E) Cell layer thickness as determined by counting nuclei at three points per field in H&E-stained sections. (F) Quantitation of ciliation by counting ciliated cells in H&E-stained sections. (G) Number of goblet cells per field in H&E-stained sections. (H) Number of vacuolated cells per field in H&E-stained sections. (I) Number of cells exhibiting pyknotic nuclei in H&E-stained sections. (D-I) Five images from each of three inserts were analysed and data displayed is mean +/- standard deviation from four animals. Statistical significance was assessed by Student’s *t*-test (D-I). Significance is indicated by one (*P*<0.05) asterisk.
(TIF)

S9 Fig Triiodothyronine (T3) does not affect differentiation of ovine tracheal epithelial cells. Ovine tracheal epithelial cells were cultured at ALI for 21 days in ALI medium with or without T3. (A) Immunofluorescent staining with anti-β-tubulin (green), rhodamine-phalloidin (red) and DAPI (blue). (B) Scanning electron microscopy. (C) Immunofluorescent staining with anti-ZO-1 (green) and DAPI (blue). (D) Haematoxylin and eosin-stained histological sections. (E) Periodic acid-Schiff-stained histological sections. (F) Anti-p63 IHC of histological sections; p63-positive cells exhibit brown nuclei. (G) Quantitation of ciliation as percentage of total area from β-tubulin staining. (H) Cell layer thickness measured from three points per field in H&E-stained sections. (I) Cell layer thickness as determined by counting nuclei at three points per field in H&E-stained sections. (J) Trans-epithelial electrical resistance measurement. Data shown are from a single representative animal with mean +/- standard deviation from three inserts displayed. (K) Quantitation of ciliation by counting ciliated cells in H&E-stained sections. (L) Number of goblet cells per field in H&E-stained sections. (M) Number of vacuolated cells per field in H&E-stained sections. (N) Number of cells exhibiting pyknotic nuclei in H&E-stained sections. (G-I, K-N) Five images from each of three inserts were analysed and data displayed is mean +/- standard deviation from four animals. Statistical significance was assessed by Student’s *t*-test (G-N). Significance values are indicated by one (*P*<0.05), two (*P*<0.01) or three (*P*<0.001) asterisks.
(TIF)
