# Supplementary figures and images for: Optimisation of growth conditions for ovine airway epithelial cell differentiation at an air-liquid interface
Source: PLoS One. 2018 Mar 8;13(3):e0193998. doi: 10.1371/journal.pone.0193998 (PMC5843276; doi:10.1371/journal.pone.0193998)

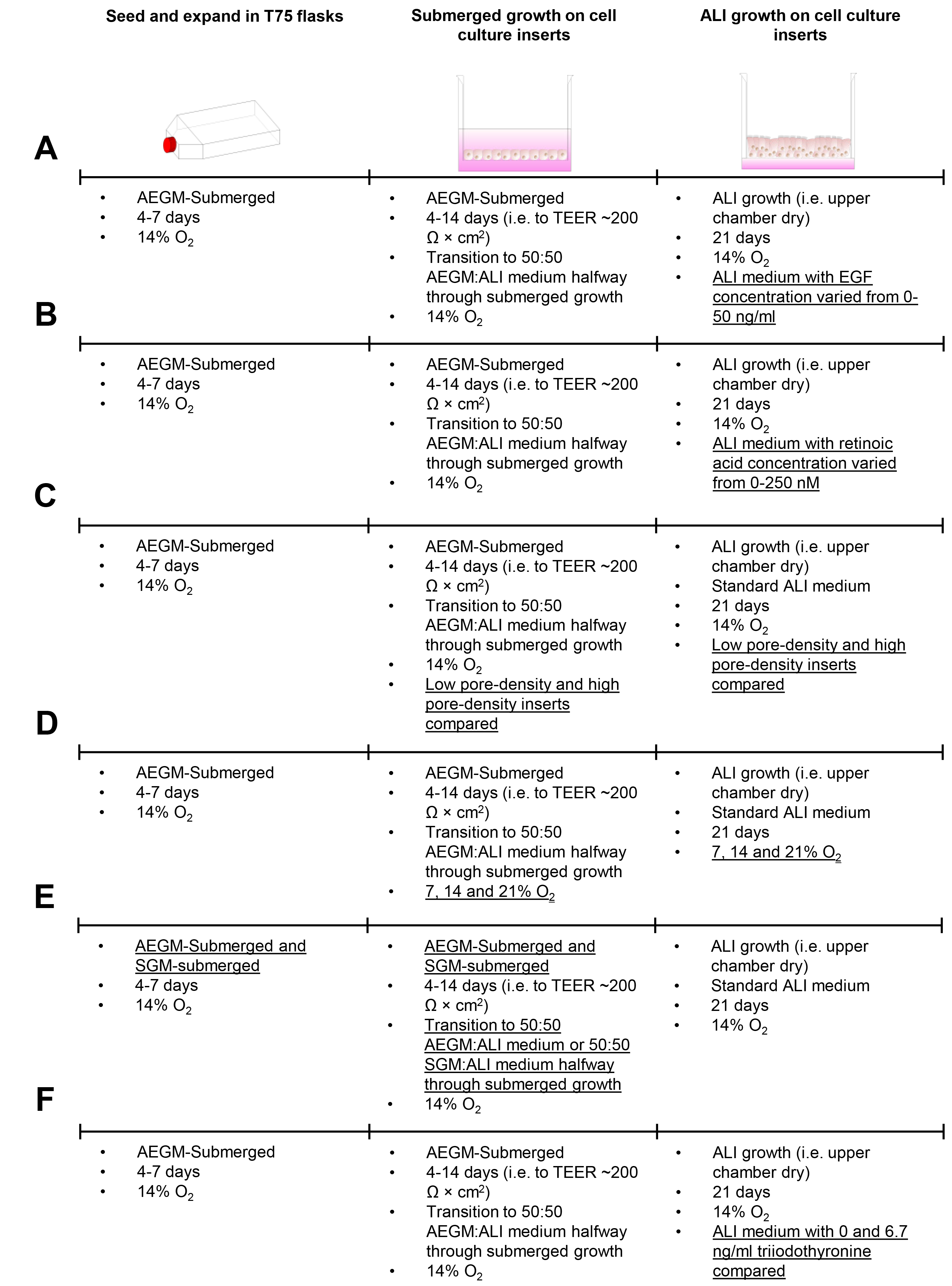

Supplement: S1 Fig — The tests carried out in this study (A-F) are outlined together with the duration of each growth phase. Specific medium formulations are detailed. The parameters which were varied in each test are underlined. (TIF) [file pone.0193998.s001.tif]

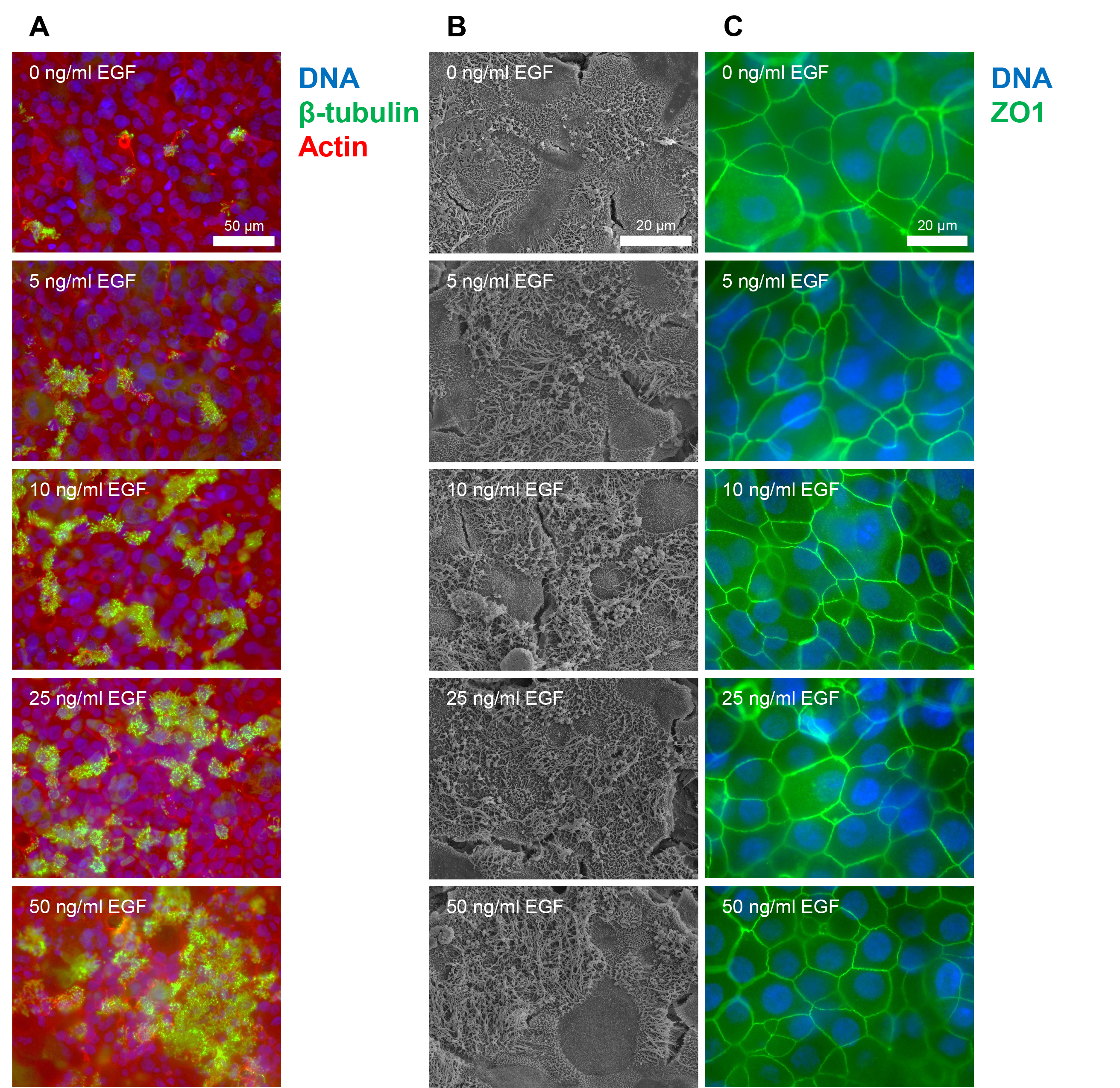

Supplement: S2 Fig — Ovine tracheal epithelial cells were cultured at ALI for 21 days with the indicated concentrations of EGF. (A) Immunofluorescent staining with anti-β-tubulin, rhodamine-phalloidin and DAPI. (B) Scanning electron microscopy. (C) Immunofluorescent staining with anti-ZO-1 and DAPI. (TIF) [file pone.0193998.s002.tif]

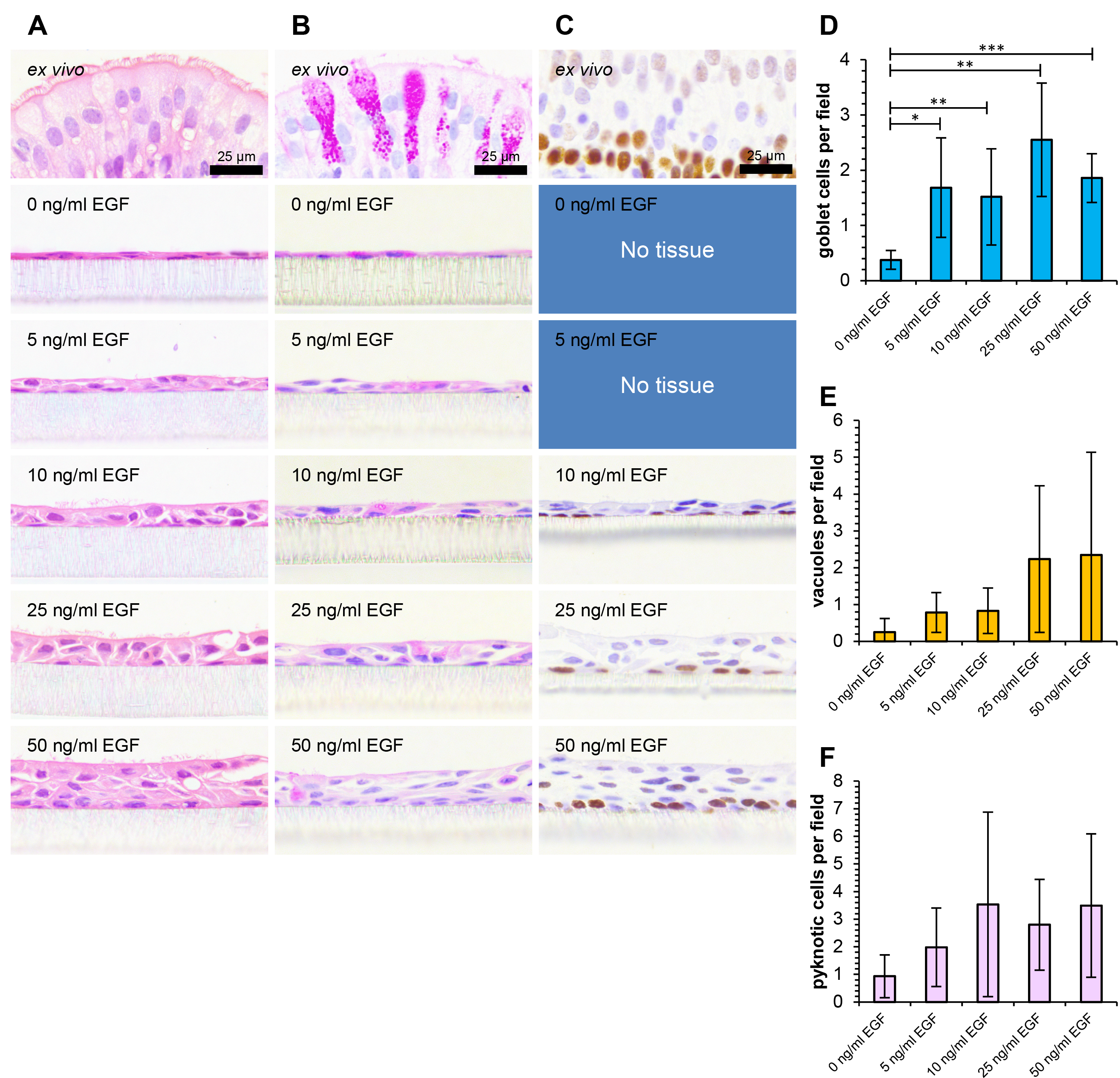

Supplement: S3 Fig — Ovine tracheal epithelial cells were cultured at ALI for 21 days with the indicated concentrations of EGF. (A) Haematoxylin and eosin-stained histological sections. (B) Periodic acid-Schiff-stained histological sections. (C) Anti-p63 IHC of histological sections; p63-positive cells exhibit brown nuclei. (D) Number of goblet cells per field in H&E-stained sections. (E) Number of vacuolated cells per field in H&E-stained sections. (F) Number of cells exhibiting pyknotic nuclei in H&E-stained sections. (D-F) Five images from each of three inserts were analysed and data displayed is mean +/- standard deviation from four animals. Statistical significance was assessed by Student’s t-test (D-F). Significance values are indicated by one (P<0.05), two (P<0.01) or three (P<0.001) asterisks. (TIF) [file pone.0193998.s003.tif]

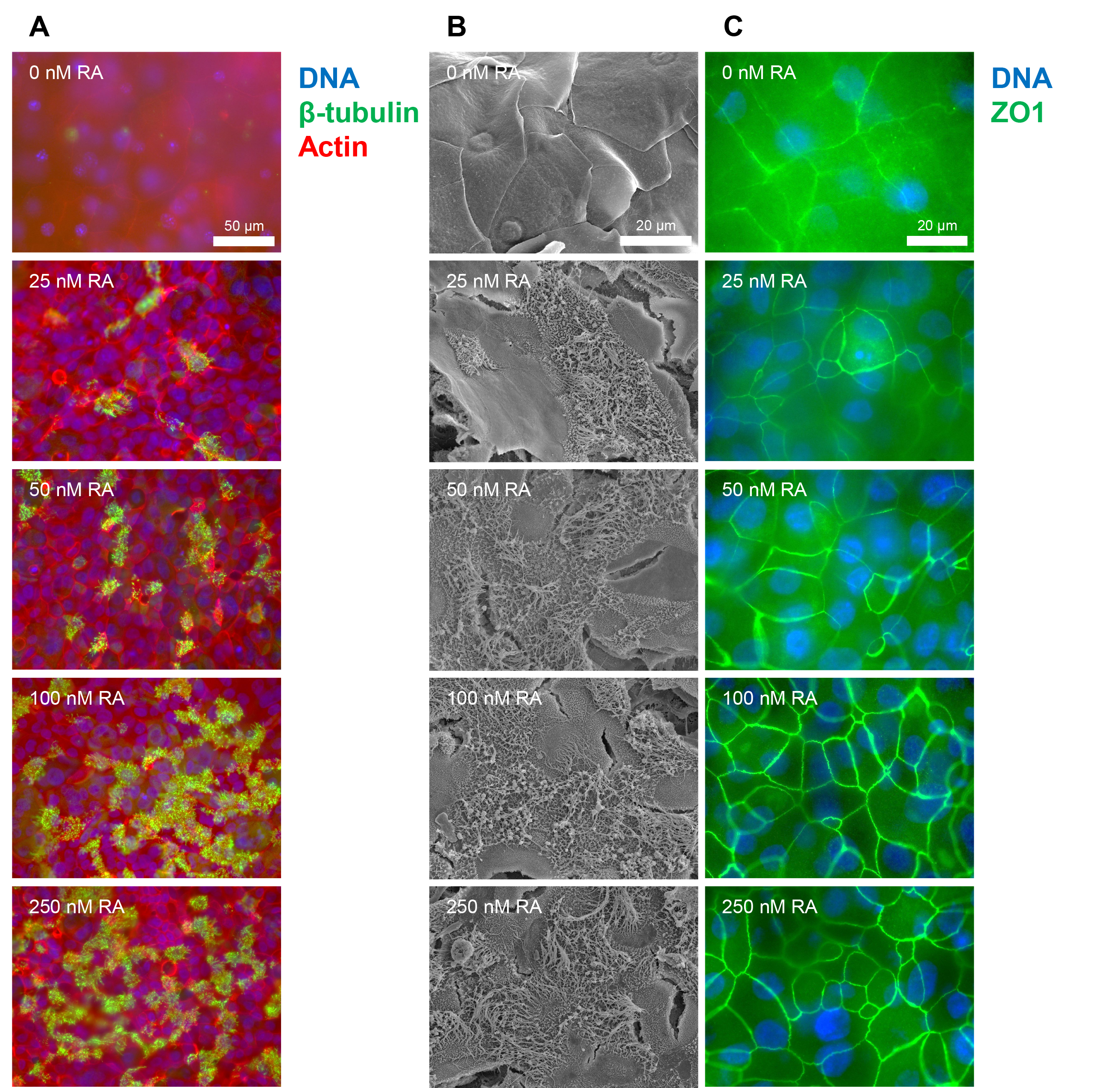

Supplement: S4 Fig — Ovine tracheal epithelial cells were cultured at ALI for 21 days with the indicated concentrations of retinoic acid. (A) Immunofluorescent staining with anti-β-tubulin, rhodamine-phalloidin and DAPI. (B) Scanning electron microscopy. (C) Immunofluorescent staining with anti-ZO-1 and DAPI. (TIF) [file pone.0193998.s004.tif]

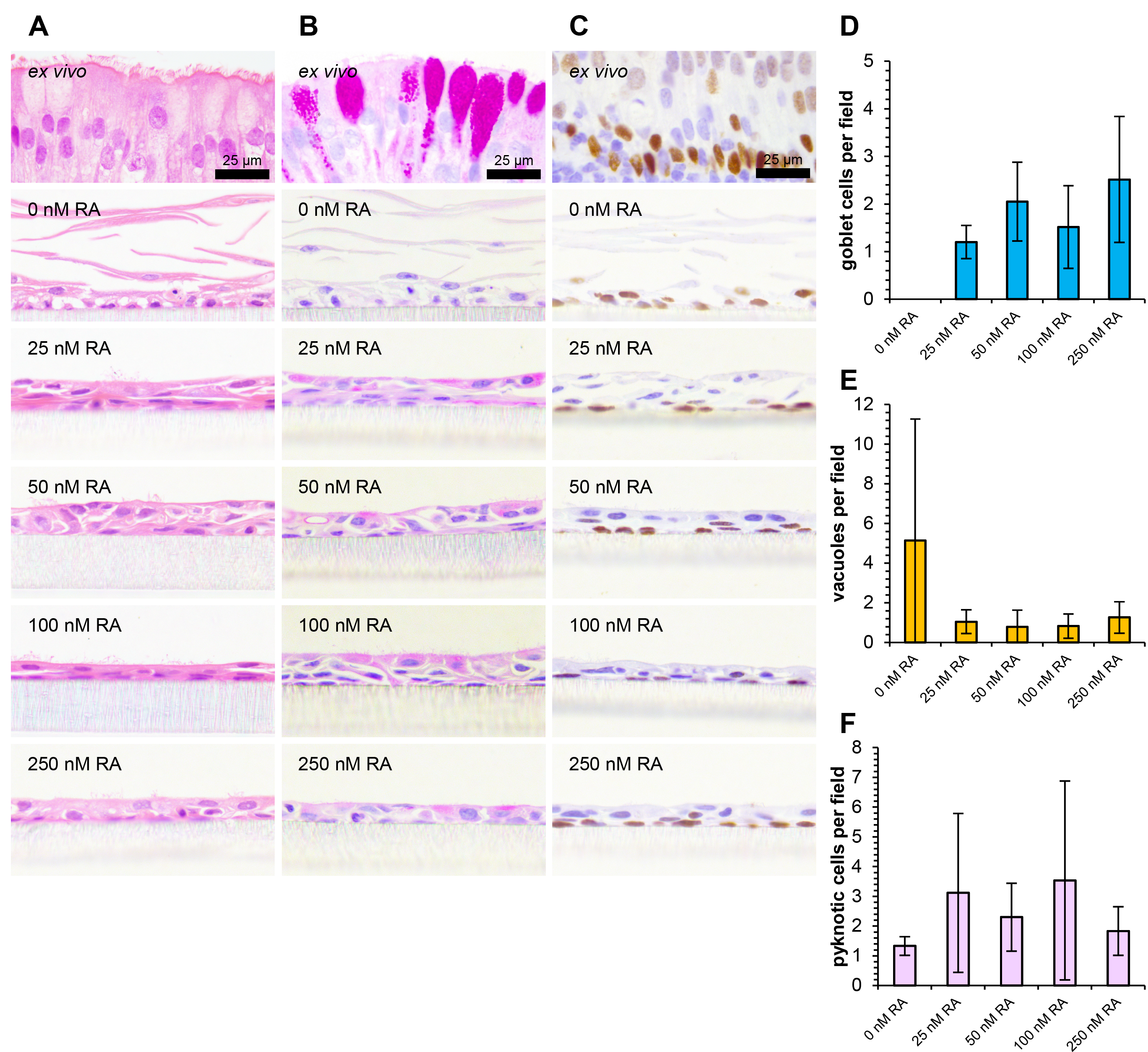

Supplement: S5 Fig — Ovine tracheal epithelial cells were cultured at ALI for 21 days with the indicated concentrations of retinoic acid. (A) Haematoxylin and eosin-stained histological sections. (B) Periodic acid-Schiff-stained histological sections. (C) Anti-p63 IHC of histological sections; p63-positive cells exhibit brown nuclei. (D) Number of goblet cells per field in H&E-stained sections. (E) Number of vacuolated cells per field in H&E-stained sections. (F) Number of cells exhibiting pyknotic nuclei in H&E-stained sections. (D-F) Five images from each of three inserts were analysed and data displayed is mean +/- standard deviation from four animals. Statistical significance was assessed by Student’s t-test (D-F). No significant differences were observed. (TIF) [file pone.0193998.s005.tif]

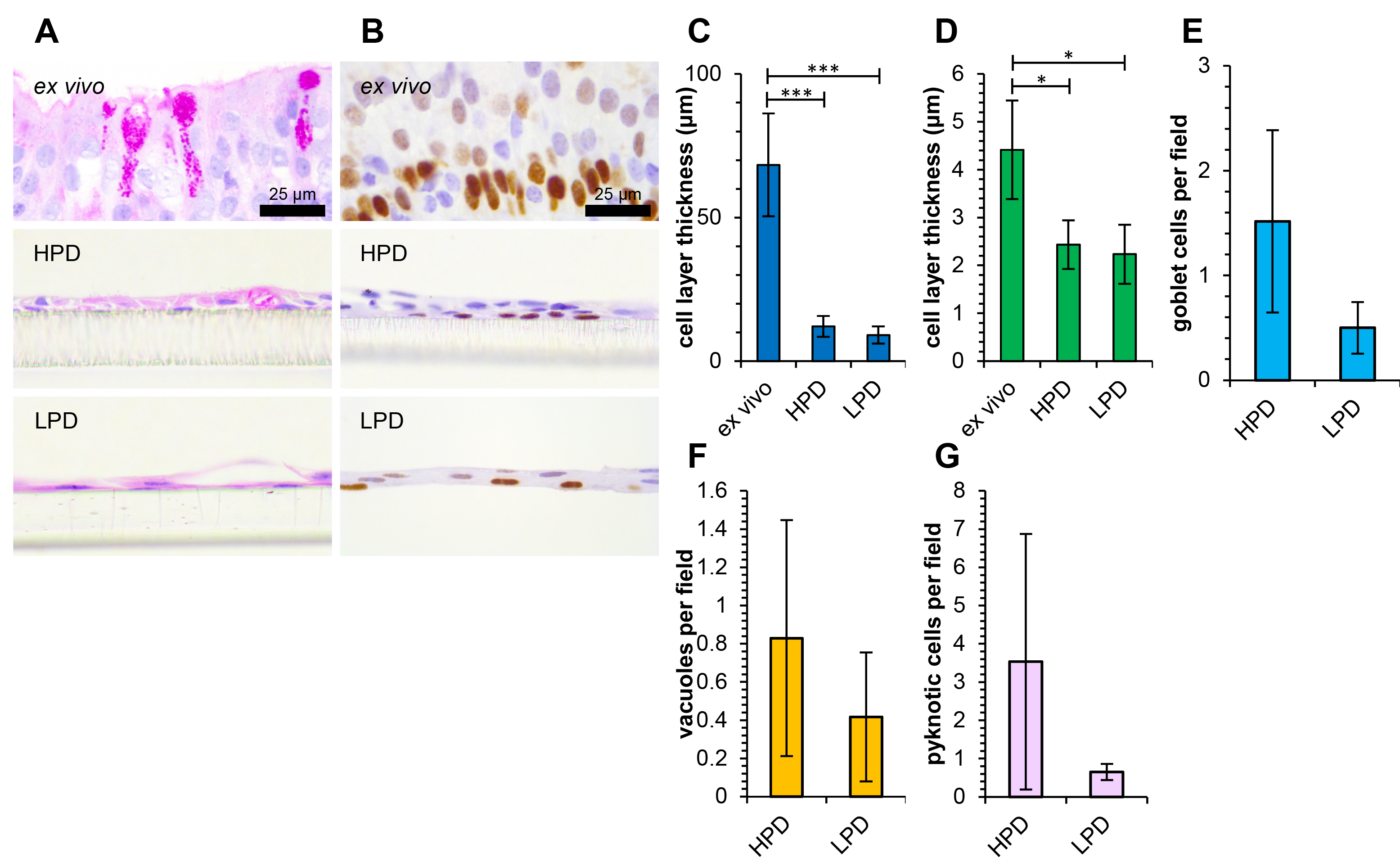

Supplement: S6 Fig — Ovine tracheal epithelial cells were cultured at ALI for 21 days on high pore-density (HPD) or low pore-density (LPD) cell culture inserts. (A) Periodic acid-Schiff-stained histological sections. (B) Anti-p63 IHC of histological sections; p63-positive cells exhibit brown nuclei. (C) Cell layer thickness measured from three points per field in H&E-stained sections. (D) Cell layer thickness as determined by counting nuclei at three points per field in H&E-stained sections. (E) Number of goblet cells per field in H&E-stained sections. (F) Number of vacuolated cells per field in H&E-stained sections. (G) Number of cells exhibiting pyknotic nuclei in H&E-stained sections. (C-G) Five images from each of three inserts were analysed and data displayed is mean +/- standard deviation from four animals. Statistical significance was assessed by Student’s t-test (C-G). Significance values are indicated by one (P<0.05) or three (P<0.001) asterisks. (TIF) [file pone.0193998.s006.tif]

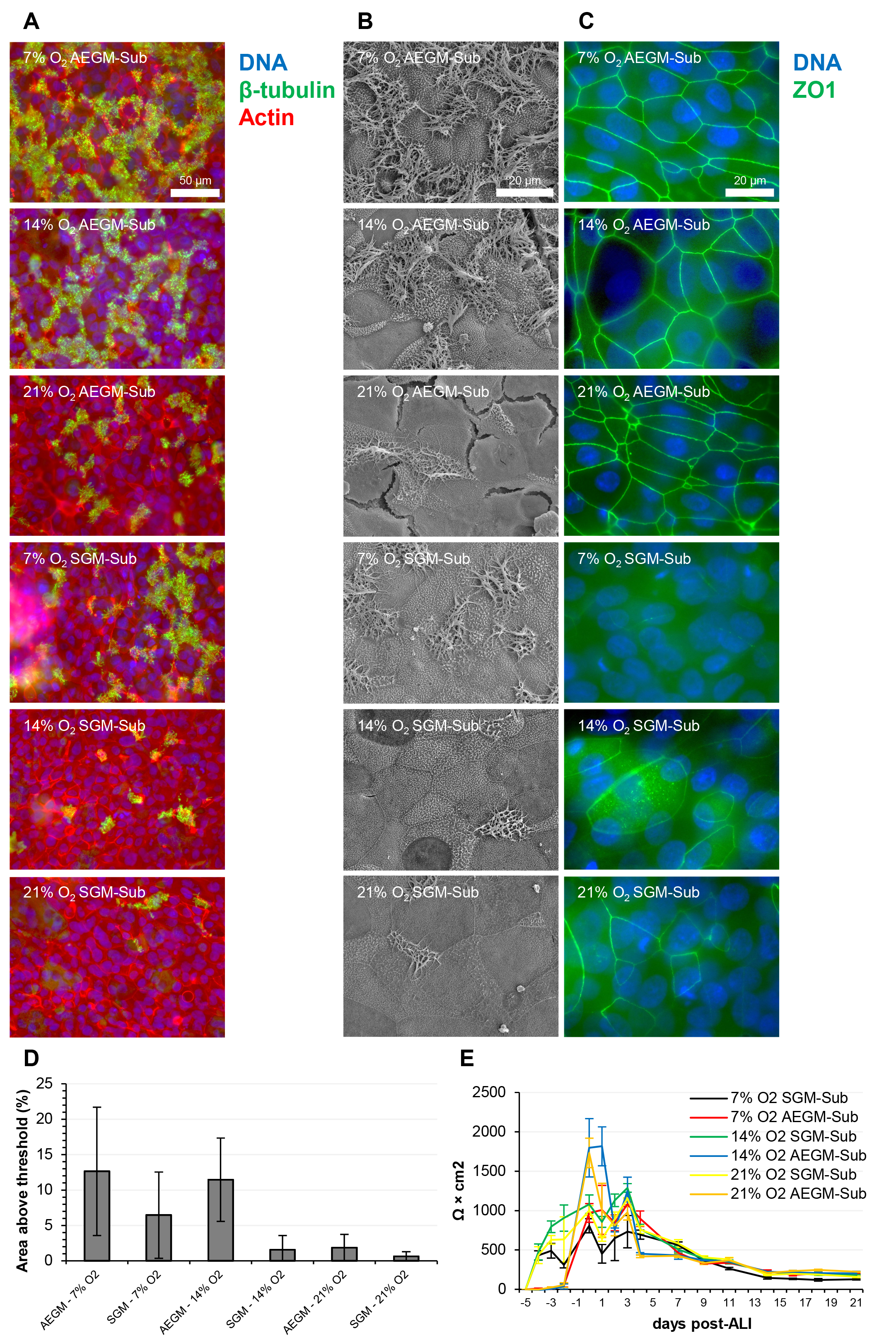

Supplement: S7 Fig — Ovine tracheal epithelial cells were cultured to confluency (while submerged) on cell culture inserts in SGM or AEGM and at ALI in ALI medium. A humidified atmosphere comprising 5% CO2 and either 7, 14 or 21% O2 was employed for submerged and ALI growth phases. (A) Immunofluorescent staining with anti-β-tubulin, rhodamine-phalloidin and DAPI. (B) Scanning electron microscopy. (C) Immunofluorescent staining with anti-ZO-1 and DAPI. (D) Quantitation of ciliation as percentage of total area from β-tubulin staining. Five images from each of three inserts were analysed and data displayed is mean +/- standard deviation from four animals. (E) Trans-epithelial electrical resistance measurement. Data shown are from a single representative animal with mean +/- standard deviation from three inserts displayed. (TIF) [file pone.0193998.s007.tif]

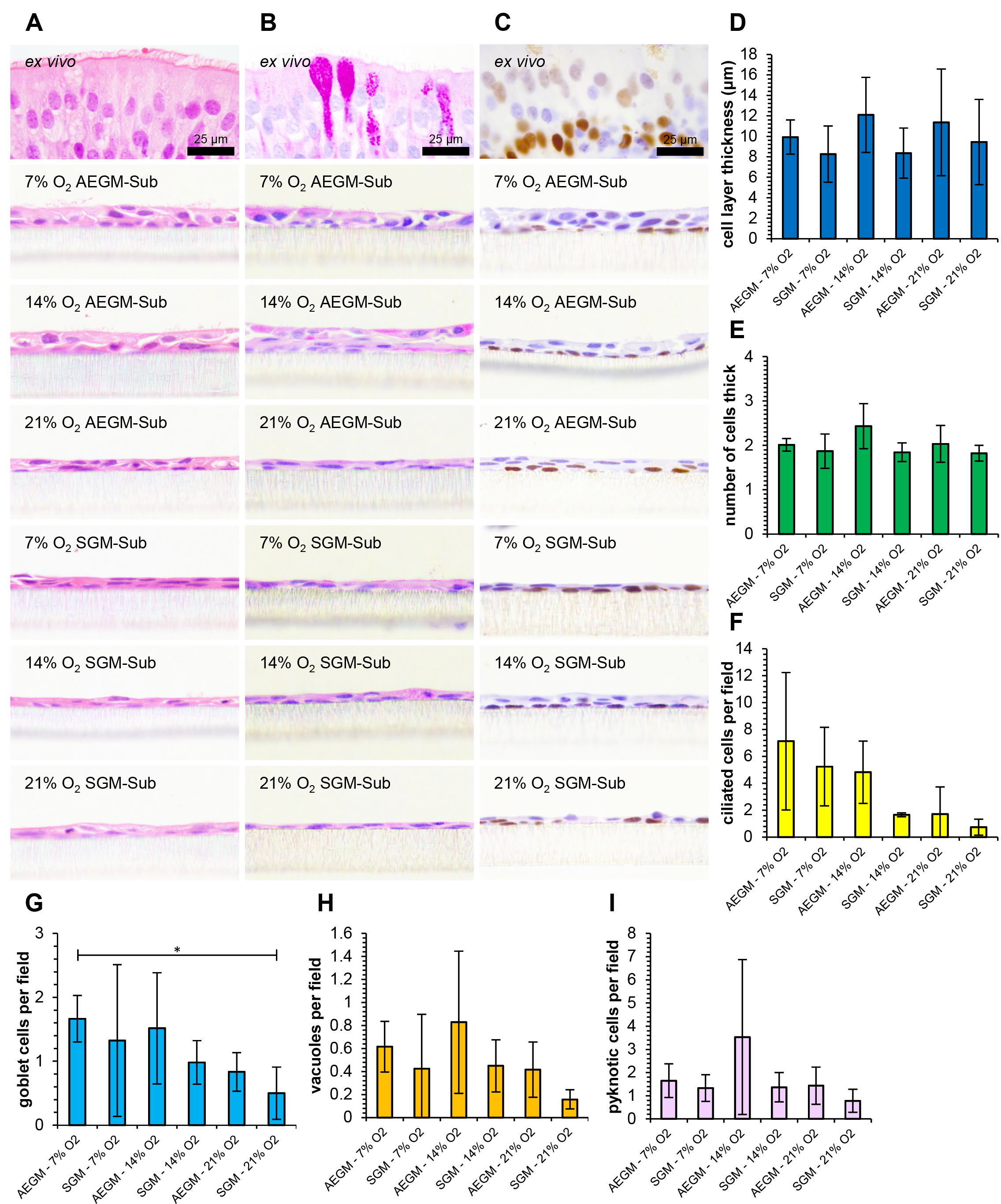

Supplement: S8 Fig — Ovine tracheal epithelial cells were cultured to confluency in SGM or AEGM and at ALI in ALI medium. A humidified atmosphere comprising 5% CO2 and either 7, 14 or 21% O2 was employed for submerged and ALI growth phases. (A) Haematoxylin and eosin-stained histological sections. (B) Periodic acid-Schiff-stained histological sections. (C) Anti-p63 IHC of histological sections; p63-positive cells exhibit brown nuclei. (D) Cell layer thickness measured from three points per field in H&E-stained sections. (E) Cell layer thickness as determined by counting nuclei at three points per field in H&E-stained sections. (F) Quantitation of ciliation by counting ciliated cells in H&E-stained sections. (G) Number of goblet cells per field in H&E-stained sections. (H) Number of vacuolated cells per field in H&E-stained sections. (I) Number of cells exhibiting pyknotic nuclei in H&E-stained sections. (D-I) Five images from each of three inserts were analysed and data displayed is mean +/- standard deviation from four animals. Statistical significance was assessed by Student’s t-test (D-I). Significance is indicated by one (P<0.05) asterisk. (TIF) [file pone.0193998.s008.tif]

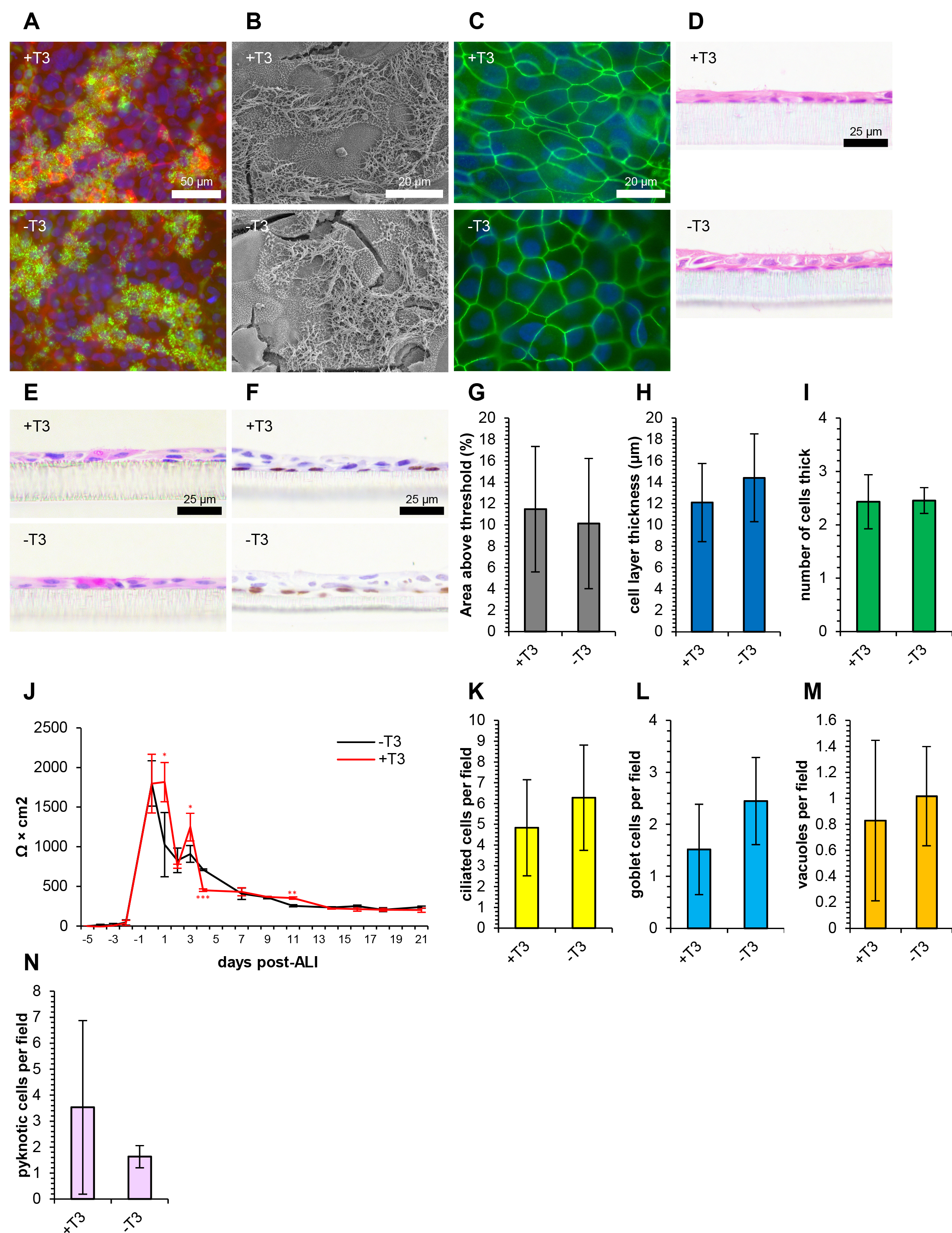

Supplement: S9 Fig — Ovine tracheal epithelial cells were cultured at ALI for 21 days in ALI medium with or without T3. (A) Immunofluorescent staining with anti-β-tubulin (green), rhodamine-phalloidin (red) and DAPI (blue). (B) Scanning electron microscopy. (C) Immunofluorescent staining with anti-ZO-1 (green) and DAPI (blue). (D) Haematoxylin and eosin-stained histological sections. (E) Periodic acid-Schiff-stained histological sections. (F) Anti-p63 IHC of histological sections; p63-positive cells exhibit brown nuclei. (G) Quantitation of ciliation as percentage of total area from β-tubulin staining. (H) Cell layer thickness measured from three points per field in H&E-stained sections. (I) Cell layer thickness as determined by counting nuclei at three points per field in H&E-stained sections. (J) Trans-epithelial electrical resistance measurement. Data shown are from a single representative animal with mean +/- standard deviation from three inserts displayed. (K) Quantitation of ciliation by counting ciliated cells in H&E-stained sections. (L) Number of goblet cells per field in H&E-stained sections. (M) Number of vacuolated cells per field in H&E-stained sections. (N) Number of cells exhibiting pyknotic nuclei in H&E-stained sections. (G-I, K-N) Five images from each of three inserts were analysed and data displayed is mean +/- standard deviation from four animals. Statistical significance was assessed by Student’s t-test (G-N). Significance values are indicated by one (P<0.05), two (P<0.01) or three (P<0.001) asterisks. (TIF) [file pone.0193998.s009.tif]
